# Integrated proteomic, phosphoproteomic, and *N*-glycoproteomic analyses of small extracellular vesicles from C2C12 myoblasts identify specific PTM patterns in ligand-receptor interactions

**DOI:** 10.1186/s12964-024-01640-8

**Published:** 2024-05-16

**Authors:** Xiulan Chen, Xi Song, Jiaran Li, Jifeng Wang, Yumeng Yan, Fuquan Yang

**Affiliations:** 1 Key Laboratory of Protein and Peptide Pharmaceuticals & Laboratory of Proteomics, Institute of Biophysics, Chinese Academy of Sciences, Beijing, 100101 China; 2 University of Chinese Academy of Sciences, Beijing, 100049 China

**Keywords:** Small extracellular vesicles (sEVs), C2C12 myoblast, Skeletal muscle, Proteomics, Phosphoproteomics, *N*-glycoproteomics, LC–MS/MS, Membrane transporters, Ligand-receptor interaction

## Abstract

Small extracellular vesicles (sEVs) are important mediators of intercellular communication by transferring of functional components (proteins, RNAs, and lipids) to recipient cells. Some PTMs, including phosphorylation and *N*-glycosylation, have been reported to play important role in EV biology, such as biogenesis, protein sorting and uptake of sEVs. MS-based proteomic technology has been applied to identify proteins and PTM modifications in sEVs. Previous proteomic studies of sEVs from C2C12 myoblasts, an important skeletal muscle cell line, focused on identification of proteins, but no PTM information on sEVs proteins is available.

In this study, we systematically analyzed the proteome, phosphoproteome, and *N*-glycoproteome of sEVs from C2C12 myoblasts with LC–MS/MS. In-depth analyses of the three proteomic datasets revealed that the three proteomes identified different catalogues of proteins, and PTMomic analysis could expand the identification of cargos in sEVs. At the proteomic level, a high percentage of membrane proteins, especially tetraspanins, was identified. The sEVs-derived phosphoproteome had a remarkably high level of tyrosine-phosphorylated sites. The tyrosine-phosphorylated proteins might be involved with EPH-Ephrin signaling pathway. At the level of *N*-glycoproteomics, several glycoforms, such as complex N-linked glycans and sialic acids on glycans, were enriched in sEVs. Retrieving of the ligand-receptor interaction in sEVs revealed that extracellular matrix (ECM) and cell adhesion molecule (CAM) represented the most abundant ligand-receptor pairs in sEVs. Mapping the PTM information on the ligands and receptors revealed that *N*-glycosylation mainly occurred on ECM and CAM proteins, while phosphorylation occurred on different categories of receptors and ligands. A comprehensive PTM map of ECM-receptor interaction and their components is also provided.

In summary, we conducted a comprehensive proteomic and PTMomic analysis of sEVs of C2C12 myoblasts. Integrated proteomic, phosphoproteomic, and *N-*glycoproteomic analysis of sEVs might provide some insights about their specific uptake mechanism.

**Supplementary Information:**

The online version contains supplementary material available at 10.1186/s12964-024-01640-8.

## Background

Extracellular vesicles (EVs) are small membrane vesicles secreted from almost all cell types. EVs contain many functional components, such as proteins, different types of RNAs, lipids, and metabolites [1, 2]. Though initially being identified as a mechanism for removal of cellular waste [3], EVs have now been identified as important mediators of intercellular communication by transferring the functional components to recipient cells [4]. EVs have been involved in a diverse range of biological processes, such as signaling transduction, antigen presentation and regulation of immune responses [5–7], as well as in some pathological conditions or diseases, such as cancer metastasis and neurodegenerative disorders [8–10].

Over the years, EVs have been broadly classified into three groups according to their physical size and biogenesis pathways: exosomes (30–200 nm), microvesicles (MVs) (100–1000 nm) and apoptotic bodies (> 1000 nm) [11]. Exosomes are formed when endosomal membrane buds inwardly to create intraluminal vesicles (ILVs), which mature into multivesicular body (MVB) and then fuse with plasma membrane to secret as exosomes. MVs are produced by outward budding followed by pinching of plasma membrane, and apoptotic bodies are released when plasma membrane blebbing occurs during apoptosis [12]. However, there are no universal molecular markers to distinguish different subtypes of EVs. In 2018, International Society for Extracellular Vesicles (ISEV) suggests classification of EV subtypes based on physical characteristics of EVs. For example, EVs can be classified into small EVs (sEVs) (< 200 nm) and medium/large EVs (> 200 nm) based on size [13]. Since sEVs (primary exosomes and to a less extent MVs in the previous nomenclature) are implicated in numerous physiological processes and diseases, we focus on this subgroup in this study.

Mass spectrometry (MS)-based proteomic technologies have been applied to characterize the molecular composition of sEVs from different tissues and cells [14]. The rapid development of MS-based proteomic technologies has enabled identification of thousands of proteins in sEVs. Besides proteins, some protein post-translational modifications (PTMs), such as phosphorylation, glycosylation, ubiquitination, sumoylation, palmitoylation, oxidation, and citrullination, have been identified in EVs [15, 16]. PTMs have been reported to play important roles in EV biology [17, 18]. For example, phosphorylation influences the biogenesis and release of EVs [19], and participates in cellular communication by transferring kinases [20] or phosphatases [21] between cells. Glycosylation has been reported to involve in biogenesis, protein sorting and uptake of EVs [22–24]. Analysis of PTMs in sEVs at proteomic level could provide more information about the roles of PTMs in sEVs.

C2C12 myoblasts, an extensively studied skeletal muscle cell line [25, 26], can secrete EVs into the culture media [27–29]. However, previous proteomic studies of C2C12 sEVs focused on the identification of proteins, no PTM information on C2C12 sEVs proteins is available.

In this study, we systematically analyzed the proteome, phosphoproteome, and *N*-glycoproteome of sEVs derived from C2C12 myoblasts. We found that the three proteomes identified distinct catalogues of proteins in sEVs. PTMomic analysis could expand the identification of cargos in sEVs. PTM modifications (phosphorylation and *N*-glycosylation) in sEVs on the ligand-receptor interactions of sEVs were extensively discussed, which might account for the targeted uptake of sEVs by recipient cells.

## Materials and methods

### Cell culture

Mouse C2C12 myoblasts were gifts from Professor Pingsheng Liu from Institute of Biophysics, Chinese Academy of Sciences. Mouse C2C12 myoblasts were maintained in DMEM medium supplemented with 10% fetal bovine serum (FBS), 100 U/ml penicillin, and 100 μg/ml streptomycin at 37℃, 5% CO_2_.

### Isolation of small extracellular vesicles (sEVs) from C2C12 myoblasts

The isolation of sEVs from C2C12 myoblasts was performed with a standard ultracentrifugation-based method described previously [30] with minor modifications. Briefly, cell-conditioned medium was collected from approximately 90% confluent C2C12 myoblasts grown for 48 h in 100 mm cell culture dishes with DMEM medium containing FBS depleted of bovine serum extracellular vesicles by ultracentrifugation at 120,000 g for 24 h. The collected cell culture medium was first subjected to centrifugation at 400 g for 10 min to pellet and remove cells. Next, the supernatant was centrifuged at 2,000 g for 20 min to remove cell debris and apoptotic bodies. Then, the supernatant was centrifuged at 15,000 g for 40 min to remove large EVs. To remove any remaining of large EVs, the supernatant from the first 15,000 g step was passed through a 0.22 mm pore PES filter (Corning). This supernatant (pre-cleared medium) was subjected to ultracentrifugation at 120,000 g for 4 h (Rotor: 45Ti, Beckman Coulter, Fullerton, CA) to sediment small EVs (sEVs). The crude sEV pellet was re-suspended in a large volume of ice-cold PBS followed by ultracentrifugation at 120,000 g for 4 h. The pellet (sEV sample) was re-suspended in 100 μl PBS supplemented with EDTA-free complete protease inhibitor cocktail and phosphatase inhibitor cocktail. All centrifugation steps were performed at 4℃. The obtained sEV samples were stored at -80℃ for less than half a year before subsequent sample preparation and PTM enrichments.

Cell culture medium from 30*100 mm cell culture dishes (~ 300 ml) was collected for one purification of sEVs. Eight purifications were performed to obtain enough sEVs proteins for further characterization of sEVs, PTM (phosphorylation and *N*-glycosylation) enrichments, and LC–MS/MS analysis.

### Transmission electron microscope (TEM) analysis

For negative staining TEM analysis, 5 µL sEV sample in PBS was loaded on a hydrophilized carbon-coated 230 mesh copper grid (Beijing Zhongjingkeyi Technology Co., China) and allowed to settle for 1 min. The sample was blotted and negatively stained with 3 continuous drops of 2% uranyl acetate, blotting between each drop. After staining the sample with the last drop for 1 min, the grid was blotted and air-dried. Grids were imaged with a Tecnai Spirit (FEI Co., US) TEM operating at 100 kV.

### Nanoparticle tracking analysis (NTA)

The concentration and size distribution of sEV samples was determined by ZetaView (Particle Matrix, German) equipped with a 488 nm laser. Samples were 1:10, 000 diluted in PBS to obtain around 300 particles/view. Three videos of 20 s duration were recorded for each independent replicate and used to compute the particle size and mean concentration. Data were analyzed with ZetaView 8.04.10 software.

### Protein extraction and western blotting

Total cell lysate (TCL) was extracted from C2C12 myoblasts as described previously [31]. Briefly, C2C12 myoblasts were collected in the lysis buffer containing 8 M urea and 100 mM Tris–HCl (pH 8.5) supplemented with EDTA-free complete protease inhibitor cocktail (Roche, Basel, Switzerland). Then, the cells were lysed with Precellys Evolution homogenizer (Bertin Technologies, Paris, France). After centrifugation at 20,000 g for 20 min at 4℃, the supernatant was collected as TCL. sEV samples were solubilized in the same lysis buffer. The protein concentration of TCL and sEVs was determined using a BCA protein assay kit (Thermo Fisher Scientific, Waltham, MA).

For western blotting, equal amounts of proteins from sEV and TCL were subjected to SDS-PAGE. After separation, proteins were transferred to Immobilon PVDF Membrane at 200 mA for 1–2 h. PVDF Membranes were blocked with 5% non-fat dry milk in TBST for 2 h at RT. After blocking, PVDF membranes were washed with TBST and incubated with primary antibodies diluted in 5% milk overnight at 4˚C. Primary antibodies used for immunoblotting were ALIX (Cat#ab186429, Abcam; 1:1,000), TSG101 (Cat# ab125011, Abcam; 1:1,000), CD9 (Cat#ab92726, Abcam; 1:1,000), HSP90 (Cat#ab203126, Abcam; 1:10,000), and HRP-conjugated GAPDH (AC035, ABclonal; 1:1,000). PVDF Membranes were then washed with TBST and incubated with secondary antibodies diluted in TBST. After washing, the secondary antibodies were detected using SuperSignal Western Blot Substrate (Thermo Fisher Scientific, Waltham, MA, USA). Four replicates of sEV samples were analyzed with western blotting.

### In-solution digestion of proteins

Three biological replicates of sEVs purified from C2C12 myoblasts were combined from eight sEV purifications and used for in-solution digestion and subsequent PTMs (phosphorylation and *N*-glycosylation) enrichment.

sEV proteins were in-solution digested into peptides as described previously [31]. In brief, proteins were reduced with 10 mM DTT at 30℃ for 1 h. The resulting free thiols were alkylated with 40 mM IAM for 45 min at room temperature in the dark. The same amount of DTT was subsequently added to remove excess IAM at 30℃ for 30 min. Then, proteins were digested with Lys-C (Wako Pure Chemical Industries, Osaka, Japan) at an enzyme/protein ratio of 1:100 (w/w) at 37℃ for 3 h. After dilution with 50 mM Tris–HCl (pH 8.0), samples were digested with sequencing grade modified trypsin (Promega, Madison, WI) at an enzyme/protein ratio of 1:50 (w/w) at 37℃ overnight. The enzymatic digestion was stopped with formic acid (FA), and the supernatant was collected after centrifugation at 20,000 g for 20 min. After that, peptides were desalted on HLB cartridges (Waters, Milford, MA, USA) and dried in SpeedVac (LABCONCO, Kansas City, MO, USA). After dissolving the desalted peptides with 0.1% FA, peptide concentration was determined using a BCA peptide assay kit (Thermo Fisher Scientific, Waltham, MA, USA). Then, peptides were split into different amounts according to different PTM enrichment experiments mentioned below and dried in SpeedVac.

### Phosphopeptide enrichment

The enrichment of phosphopeptides using TiO_2_ with lactic acid was performed as described previously [31]. Briefly, 200 μg dried peptides were re-solubilized in 100 μl sample loading buffer containing 70% ACN, 5% TFA, and 20% lactic acid (Sigma) to a final concentration of 2 μg/μl. TiO_2_ beads (5 μm Titansphere, GL Sciences, Tokyo, Japan) were preconditioned with sample loading buffer for 5 min, and the process was repeated three times. Subsequently, the peptides were incubated with preconditioned TiO_2_ beads at a peptides/TiO_2_ ratio of 1:6 (w/w) for 15 min at room temperature. After pelleted TiO_2_ beads, the supernatant was transferred to another tube and incubated with half of the amount of TiO_2_ beads used in the first incubation. A third incubation was performed with 1/4 of the amount of TiO_2_ beads used in the first incubation. TiO_2_ beads from the three incubations were pooled with loading buffer and transferred to preconditioned C8 StageTips. The TiO_2_ beads were sequentially washed with sample loading buffer, washing buffer 1 (30% ACN, 0.5% TFA), and washing buffer 2 (80% ACN, 0.4% TFA). Phosphopeptides on TiO_2_ beads were eluted with elution buffer 1 (4% NH_3_.H_2_O) and elution buffer 2 (4% NH_3_.H_2_O and 50% ACN) sequentially. The eluted phosphopeptides were immediately acidified with 10% FA and dried in SpeedVac. Before LC–MS/MS analysis, phosphopeptides were desalted with homemade OLIGO^TM^ R3 StageTips.

Three biological replicates of sEVs samples with two technical replicates of phosphopeptide enrichment and six LC–MS/MS analyses were performed to obtain the phosphoproteome of sEVs.

### N-glycopeptide enrichment

100 μg dried peptides were re-solubilized in 50 μl loading buffer (80% ACN, 1% TFA) before enrichment of glycopeptides with home-made ZIC-HILIC tips. ZIC-HILIC beads (Agela Technologies, China) were loaded onto 200 μl pipette tips with C8 3M membrane to make ZIC-HILIC tips. The ZIC-HILIC tips were sequentially conditioned with ACN, 0.1% TFA, and 80% ACN/0.1% TFA, and peptides were loaded onto ZIC-HILIC tips. After washed ZIC-HILIC tips with 1% 80% ACN/0.1%TFA, the enriched *N*-glycopeptides were eluted with 0.1% TFA and dried for LC–MS/MS analysis.

Three biological replicates of sEVs samples with two technical replicates of *N*-glycopeptide enrichment and six LC–MS/MS analyses were performed to obtain the *N*-glycoproteome of sEVs.

### LC–MS/MS analysis

Peptides and phosphopeptides of sEVs were analyzed on an Easy-nLC 1200 HPLC system (Thermo Fisher Scientific) coupled to an Orbitrap Exploris 480 (Thermo Fisher Scientific) with a high-field asymmetric waveform ion mobility spectrometry (FAIMS) device (Thermo Fisher Scientific). All samples were reconstituted in 0.1% FA and separated on a fused silica trap column (100 μm ID * 2 cm) in-house packed with reversed-phase silica (Reprosil-Pur C18 AQ, 5 μm, Dr. Maisch GmbH, Baden-Wuerttemberg, Germany) coupled to an analytical column (75 μm ID * 20 cm) packed with reversed-phase silica (Reprosil-Pur C18 AQ, 3 μm, Dr. Maisch GmbH). The peptides and phosphopeptides were analyzed with 103 min gradient (buffer A: 0.1% FA in H_2_O, buffer B: 80% ACN, 0.1% FA in H_2_O) at a flow rate of 300 nL/min (5–9% B, 4 min; 9–20% B, 32 min; 20–30% B, 31 min; 30–40% B, 23 min; 40–99% B, 4 min; 99% B, 9 min). MS data were acquired using an Orbitrap mass analyzer in data-dependent acquisition mode. The cycle time was set as 2 s. The spray voltage of the nanoelectrospray ion source was 2.0 kV, and the heated capillary temperature was 320℃. Full scan MS data were collected at a high resolution of 60,000 (m/z 200) from 350 to 1500 m/z. The automatic gain control target was 4*10^5^, dynamic exclusion was 30 s, and the intensity threshold was 5.0*10^4^. The precursor ions were selected from each MS full scan with an isolation width of 1.6 m/z for fragmentation with a normalized collision energy of 30%. For phosphopeptide analysis, MS/MS data were acquired at a resolution of 30,000 (m/z 200). The automatic gain control target was 1*10^5^, the maximum injection time was 54 ms, dynamic exclusion was 30 s, and the intensity threshold was 5.0*10^4^. For peptide analysis, MS/MS data were acquired at a resolution of 15,000 (m/z 200). The automatic gain control target was 5*10^4^; the maximum injection time was 22 ms. The compensation voltage of FAIMS was set as -45 V and -65 V.

The enriched *N*-glycopeptides of sEVs were analyzed on an Easy-nLC 1200 HPLC system (Thermo Fisher Scientific) coupled to an Orbitrap Eclipse Tribrid mass spectrometer (Thermo Fisher Scientific). *N*-glycopeptides were separated with trap column and analytical column mentioned above. The *N*-glycopeptides were analyzed with 103 min gradient at a flow rate of 300 nL/min (0–11% B, 4 min; 11–22% B, 32 min; 22–32% B, 31 min; 32–42% B, 23 min; 42–95% B, 3 min; 95% B, 10 min). MS data were acquired in data-dependent acquisition mode. The cycle time was set as 3 s. The spray voltage of the nano-electrospray ion source was 2.0 kV and the heated capillary temperature was 320 °C. An MS1 scan was acquired from 350 to 2000 m/z (60,000 resolution, 4e^5^ AGC) followed by stepped energy HCD MS/MS acquisition of the precursors and detection in the Orbitrap (30, 000 resolution, 5e^4^ AGC, maximum injection time = 100 ms, stepped collision energy = 25%, 35%, 45%).

### MS database searching

For protein or phosphopeptide identification, LC–MS/MS raw data were processed with Proteome Discoverer (PD) (version 2.4.1.15) using SequestHT search engine. The precursor detector node in PD 2.4 was added to reduce the influence of chimeric spectra. The database was UniProt reviewed mouse protein database (updated July 2022) with 17,119 entries and common contaminants. Database searching parameters were set as following: enzyme specificity for trypsin and up to two missed cleavages were allowed, minimum peptide length was 6, and mass tolerance for precursor and fragment ions were set as 10 ppm and 0.02 Da, respectively. Cysteine carbamidomethylation was set as a fixed modification. For peptide identification, methionine oxidation and acetylation at the N-terminal of proteins were set as variable modifications. For phosphopeptide identification, phosphorylation at serine, threonine, tyrosine was also set as variable modifications besides the mentioned two variable modifications. The false discovery rate (FDR) was calculated using Percolator algorithm provided by PD. FDR on peptide and protein levels was 1%. PhosphoRS localization probability for phosphopeptides was set to greater than 0.75[32]. Only phosphopeptides with fully-localized sites were regarded as localized phosphopeptides. The number of non-redundant localized phosphopeptides and localized phosphosites were extracted with an in-house python script. The contaminating proteins were excluded from further data analysis.

For *N*-glycopeptide identification, LC-MS/MS raw data were searched with the same database mentioned above using PTMcentric search engine Byonic (version 3.11.3, Protein Metrics) incorporated in PD 2.2. Trypsin was selected as the enzyme and up to two missed cleavages were allowed. Searches were performed with a precursor mass tolerance of 10 ppm and a fragment mass tolerance of 0.02 Da. Cysteine carbamidomethylation was set as static modification. Dynamic modifications included oxidation of methionine residues, deamidation of asparagine and glutamine, and *N*-glycosylation on asparagine. Oxidation and deamidation were set as “rare” modifications, and *N*-glycosylation was set as “common” modification. Two rare modifications and one common modification were allowed. Mammalian N-glycan database embedded in Byonic, which contains 309 glycan entities, was used. Results were filtered to 1% protein FDR as set in Byonic parameters, and data was further processed to 1% FDR at the PSM level using the 2D-FDR score (a simple variation of the standard target-decoy strategy that estimates and controls PSM and protein FDRs simultaneously) [33, 34]. Only *N*-glycopeptides with Byonic score > 100 and |logProb|> 1 were reported (the absolute value of the log base 10 of the protein *p*-value). Each *N*-glycopeptide identified should have the consensus motif N-X-S/T (X ≠ P). This filtering criteria has been reported to result in confident glycosite assignment at glycopeptide spectral match level [35]. The contaminating proteins were excluded from further data analysis.

### Gene ontology and pathway analysis of three proteomic data

Gene ontology (GO) analysis of three proteomic data (proteome, phosphoproteome, and *N*-glycoproteome) was performed with Panther GOSlim[36], DAVID Bioinformatics Resource [37], and ToppCluster [38]. The hypergeometric statistical test and Benjamini & Hochberg false discovery rate correction were adopted to derive overrepresented functions. The level of significance was set as P ≤ 0.05. Pathway analysis of the three proteomic data was conducted with KEGG database [39] and Reactome database [40]. ECM-receptor interaction network was retrieved from Pathview web (https://pathview.uncc.edu/) [41]. Uniprot database was used to annotate membrane proteins in the proteome of C2C12 sEVs. TOPCONS (https://topcons.cbr.su.se/) [42] was used to predict the number of transmembrane helices (TMs) of the multi-pass membrane proteins.

### Phosphoproteomic analysis of C2C12 sEVs

Localized phosphosites were mapped in PhosphoSitePlus [43] and dbPTM [44]. GPS5.0 was used to predict kinases responsible for tyrosine phosphosites. The classification of kinases identified in the three proteomic data was performed according to mouse Kinome database [45].

### Membrane transporters analysis

Membrane transporters identified in the three proteomic data were classified according to the Transporter Classification database (TCDB) [46]. PTM information was integrated on membrane transporters and presented with circular barplot.

### Ligand-receptor interaction analysis

Ligand-receptor interaction pairs in the three proteomic data of sEVs were retrieved from CellTalkDB (http://tcm.zju.edu.cn/celltalkdb), a manually curated comprehensive database of ligand-receptor interaction pairs in humans and mice [47]. The classification of ligands and receptors was on the basis of annotation of Uniprot database. The ligand-receptor interactions were constructed with Hierarchical Edge Bundling R Graph (https://r-graph-gallery.com/310-custom-hierarchical-edge-bundling.html). PTM information was integrated on the ligands and receptors with Adobe illustrator.

## Results

### Characterization of C2C12 myoblasts-derived sEVs

**Fig. 1:**
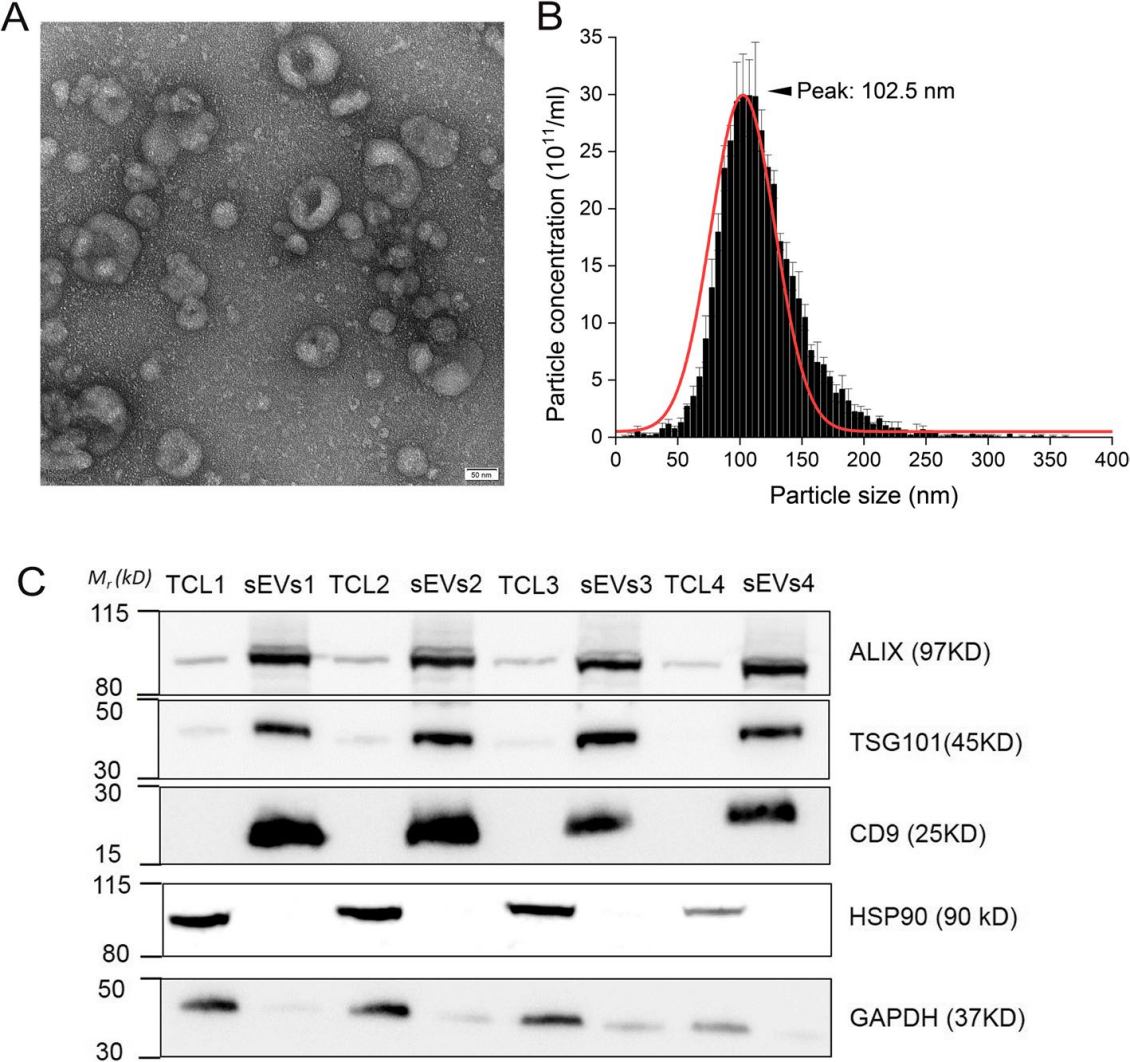
The characterization of C2C12 myoblasts-derived sEVs. **A** TEM image of sEVs isolated from the culture medium of C2C12 myoblasts. The scale bar represents 50 nm. **B** NTA results of C2C12 myoblasts-derived sEVs. The histogram represents the particle size distribution. **C** Western blot analysis of protein level in the TCL and sEVs of C2C12 myoblasts. Four replicates of sEVs and TCL proteins were analyzed. TCL, total cell lysate

To isolate sEVs from C2C12 myoblasts, cells were incubated in EVs-free FBS medium for 48 h and sEVs were isolated from cell-conditioned medium by a standard ultracentrifugation-based method [30]. Then, the morphology, size feature, and purity of C2C12 myoblasts-derived sEVs were characterized with different techniques. First, the morphology of sEVs was evaluated by TEM, which showed that the purified vesicles were membrane bound, round and heterogeneous in size (Fig. 1A). NTA result showed that the size of sEVs distributed around 100 nm with center at 102.5 nm, and the majority of sEVs were sized < 200 nm in diameter (Fig. 1B), which is consistent with previous observed size of exosomes from C2C12 myoblasts [48]. Western blotting analysis revealed that sEVs marker proteins (ALIX, TSG101, and CD9) were enriched in the sEVs samples compared with that of cells. HSP90 was exclusively present in cells and the abundance of GAPDH was remarkably higher in cells than that of sEVs (Fig. 1C). These results demonstrated a selective enrichment of sEVs from C2C12 myoblasts cell culture conditioned media.

### Proteomic analysis of sEVs from C2C12 myoblasts

To characterize the cargos in C2C12 myoblasts-derived sEVs, we conducted a comprehensive analysis of proteins, phosphoproteins, and *N*-glycoproteins with LC–MS/MS. Proteins from three biological replicates of sEVs samples were digested into peptides. For each biological replicate, two technical replicates of LC–MS/MS analyses were performed. In this way, six LC–MS/MS data were obtained for each proteomic or PTMomic analysis.

**Fig. 2:**
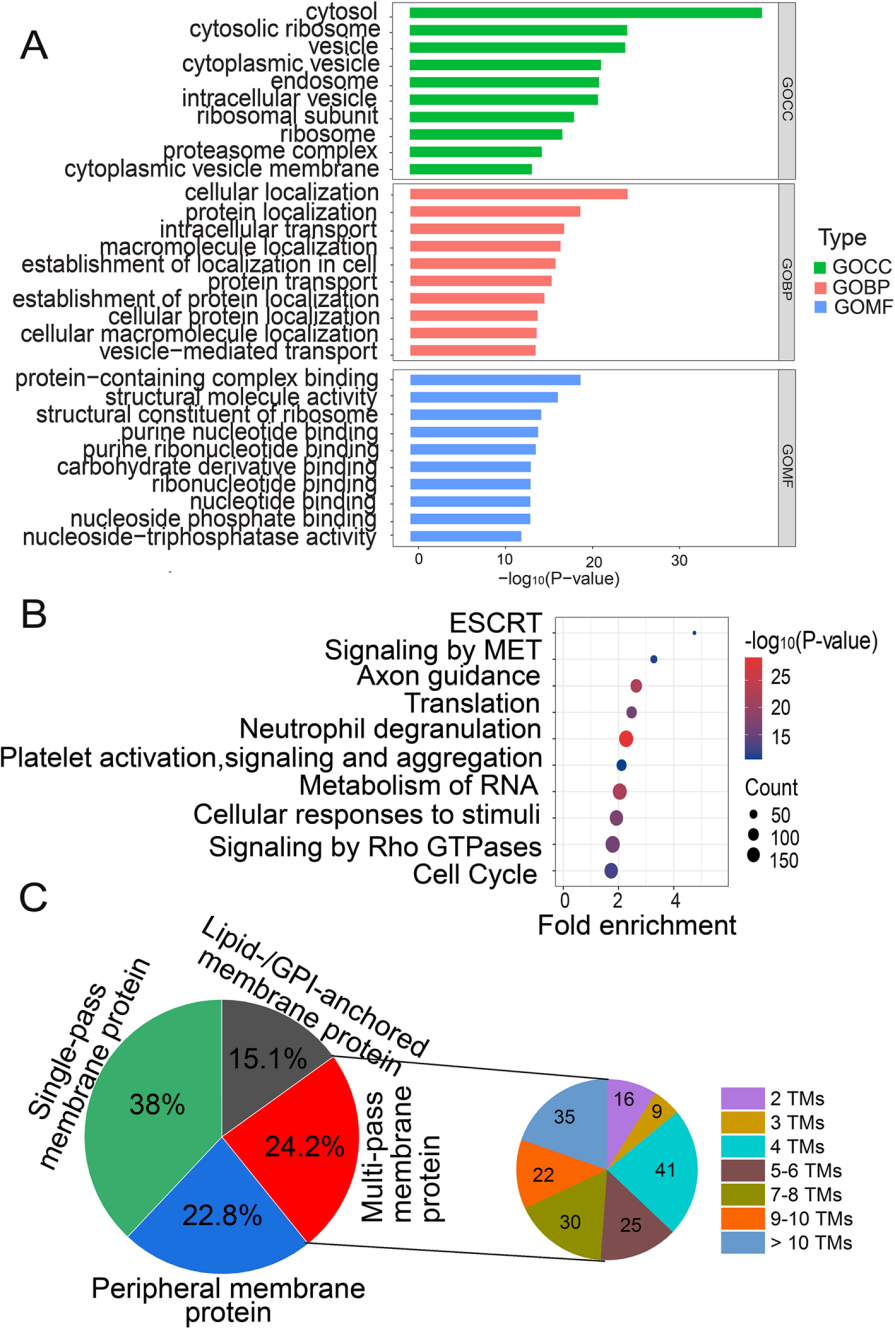
Proteomic analysis of C2C12 myoblasts-derived sEVs. **A** Analysis of the proteome of sEVs with GOCC, GOBP, and GOMF. **B** Reactome pathway analysis of the proteome of sEVs. **C** The classification of membrane proteins identified in sEVs. TMs, transmembrane helices

At the proteome level, 2024 proteins were identified in C2C12 sEVs (Table S1). sEVs proteins were categorized with GO biological process (GOBP), GO cellular component (GOCC), and GO molecular function (GOMF) using GOSlim in Panther (Fig. 2A). GOBP overrepresentation analysis revealed that sEVs proteins were enriched with vesicle structure- and vesicle biogenesis-related biological processes, such as cellular localization, protein localization, intracellular transport, protein transport, and vesicle-mediated transport, which is consistent with the role of sEVs as a means of transport. Cytosol, vesicle, and endosome were the most highly represented terms in GOCC overrepresentation analysis of sEVs proteins. Certain functional activities, such as binding activities (protein-containing complex binding, nucleotide binding), were overrepresented in sEVs, suggesting that sEVs exert their functions with binding with other functional molecules.

Top10 Reactome pathways in the proteome of sEVs are shown in Fig. 2B. Endosomal sorting complexes required for transport (ESCRT), signaling by MET, and axon guidance were the most represented pathways in sEVs. ESCRT protein complex is required for both the formation of MVB vesicles and the sorting of cargos into vesicles [49], while MET signaling and axon guidance are signaling pathways specifically reported in sEVs of C2C12 myoblasts.

As we have published a comprehensive proteomic and phosphoproteomic analysis of C2C12 myoblasts, in which 7827 proteins were identified [50], we compared the proteomes of cells and sEVs of C2C12 myoblasts. First, the number of proteins identified in sEVs was about 1/4 of that of cells, which suggested that a specific subpopulation of proteins from the cells of origin is sorted into sEVs. Second, most of proteins (1757/2024 proteins) identified in sEVs proteome were also identified in the proteome of cells, suggesting that the abundance of proteins, instead of types of proteins, accounts for the major difference of the proteomes of sEVs and cells. Furthermore, 267 proteins were exclusively identified in the proteome of sEVs (Figure S1A). GOCC analysis of these sEVs-specific proteins revealed that they were mainly from extracellular region, extracellular space, membrane, and cell surface (Figure S1B), indicating that sEVs were enriched of secreted proteins and membrane proteins. Then, we compared the proteomes of cells and sEVs with GO. GOBP analysis showed that different biological processes were overrepresented in the cells and sEVs. Vesicle structure-and vesicle biogenesis-related biological processes were overrepresented in sEVs (Fig. 2A), while different metabolic processes and organelle organization were overrepresented in the proteome of cells (Figure S1C). For GOCC analysis, cytosol and vesicles were overrepresented in sEVs (Fig. 2A), while organelle, cytoplasm, and nucleus were overrepresented in the proteome of cells (Figure S1C). These results indicated that sEVs contain a specific subpopulation of proteins, which might play specific functions for sEVs.

Since sEVs enriched of secreted proteins, we predicted classical secreted proteins in the proteome of sEVs with the workflow described previously [51]. 200 classical secreted proteins were identified in the proteome of sEV (Table S2). Functional analysis of these secreted proteins revealed that they were mainly involved in extracellular matrix organization, cell adhesion, and collagen fibril organization. They also participated in pathways of regulation of insulin-like growth factor transport and uptake by IGFBPs, and post-translational protein phosphorylation (Figure S1D), suggesting that these secreted proteins take part in signaling through post-translational protein phosphorylation.

As membrane proteins have important functions in numerous cellular processes, such as signal transduction, cell-to-cell interaction, cell-to-matrix interaction, membrane trafficking, and transmembrane transport of ions, metabolites and proteins, we annotated the proteome of sEVs with Uniprot database, and found that there was a remarkable number of membrane proteins. 737 proteins were annotated as membrane proteins (Table S3), accounting for 36.4% of the total proteins identified in sEVs. This percentage is equivalent to the estimated percentage of membrane proteins in cell culture medium-derived EVs (34%) [52].

According to intramolecular arrangement and position in the cell, membrane proteins are generally classified into six types: single-pass type I membrane protein, single-pass type II membrane protein, multi-pass membrane protein, lipid-anchored membrane protein, glycosylphosphatidylinositol (GPI)-anchored membrane protein, and peripheral membrane protein [53]. We classified the 737 membrane proteins into four types according to the annotation in Uniprot database: single-pass membrane protein, lipid-/GPI-anchored membrane protein, peripheral membrane protein, and multi-pass membrane protein. The percentage of single-pass membrane proteins, peripheral membrane proteins, multi-pass membrane proteins, and lipid-/GPI-anchored membrane proteins of all membrane proteins is 38%, 23%, 24%, and 15%, respectively (Fig. 2C). The high percentage of GPI-anchored proteins is in agreement with previous observation that GPI-anchored proteins are enriched in exosomes/EVs [54]. These proteins might perform or mediate diverse cellular functions of EVs, such as signal transduction and cell adhesion [55]. Then, we predicted TMs of the multi-pass membrane proteins and found that about 1/4 multi-pass membrane proteins had four TMs (Fig. 2C), including some tetraspanins (TSPANs) (CD9, CD63, CD81, CD151, Tspan 2–9, Tspan 14–15). The enrichment of TSPANs in sEVs probably because TSPANs can form TSPAN-complexes/ TSPAN web with other TSPANs, integrins or signaling receptors, which locate in TSPAN-enriched microdomain, and play important roles in biogenesis of exosomes, sorting proteins into sEVs, and contribute to target cell selection and uptake [56, 57].

In summary, the proteome of C2C12 sEVs is different from that of cells on the basis of GO and pathway analysis. A high percentage of membrane proteins was identified in sEVs, which is in agreement with the concept that sEVs share the same plasma membrane with their parent cells.

### Phosphoproteomic analysis of sEVs from C2C12 myoblasts

**Fig. 3:**
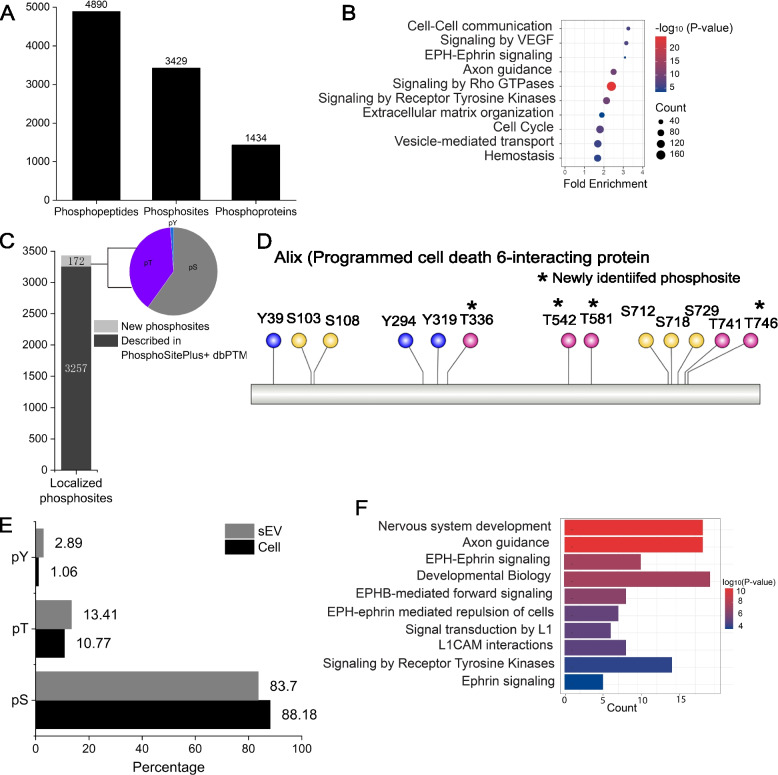
Phosphoproteomic analysis of C2C12 myoblasts-derived sEVs. **A** Phosphoproteomic results of sEVs of C2C12 myoblasts. **B** Reactome pathway analysis of phosphoproteins identified in sEVs. **C** The number and distribution of newly-identified phosphosites in sEVs. **D** Phosphosites identified on Alix, a marker protein of sEVs. **E** Comparison of the distribution of phosphosites in sEVs and cells. **F** Reactome pathway analysis of tyrosine-phosphorylated proteins in sEVs

We performed phosphoproteomic analysis of sEV samples with six LC–MS/MS analyses. 4890 phosphopeptides (including 4088 phosphopeptides with localized phosphosites), 3429 unique phosphosites, and 1434 phosphoproteins were identified in sEVs of C2C12 myoblasts (Fig. 3A; Table S4). The in-house python script for phosphoproteomic analysis is also provided in Table S4.

First, we compared the phosphoproteomes of sEVs and cells [50]. The overlap of phosphoproteins with localized phosphosites in C2C12 cells and sEVs was high. About 70% phosphoproteins identified in sEVs were also identified in cells (Figure S2A), which is similar to the proteomes of sEVs and cells. However, 409 phosphoproteins were specifically identified in the phosphoproteome of sEVs. GOCC analysis revealed that these sEV-specific phosphoproteins mainly localized in membrane, endosome, and cell surface (Figure S2B). GOBP analysis of the phosphoproteomes of sEVs and cells showed that distinct biological processes were enriched in sEVs and cells. Biological processes of protein phosphorylation, cell migration, endocytosis, and cell adhesion were enriched in the phosphoproteome of sEVs, while biological processes of chromatin, cell cycle, mRNA processing, and RNA splicing were overrepresented in the phosphoproteome of cells (Figure S2C). Cytoplasm, membrane, endosome, and plasma membrane were the most highly represented GOCC terms in the phosphoproteome of sEVs, while nucleus, cytoplasm, cytosol, and cytoskeleton were enriched in the phosphoproteome of cells (Figure S2D). Focal adhesion, proteoglycans in cancer, endocytosis, axon guidance, and regulation of actin cytoskeleton were among the top 10 enriched KEGG pathways in the phosphoproteome of sEVs, while spliceosome, proteoglycans in cancer, adherens junction, and cell cycle were among the top 10 enriched KEGG pathways in the phosphoproteome of cells (Figure S2E). Reactome pathway analysis of phosphoproteins in sEVs revealed that cell–cell communication, signaling by VEGF, EPH-Ephrin signaling, axon guidance, signaling by Rho GTPases, and signaling by Rho receptor tyrosine kinases were enriched in sEVs (Fig. 3B), suggested that phosphoproteins in sEVs might play specific roles in different signaling pathways in C2C12 myoblasts.

Detailed analysis of phosphoproteins revealed that about half of phosphoproteins in sEVs had at least two phosphosites (Figure S3A). Some important phosphoproteins in sEVs were identified with a high number of phosphosites. For example, 25 phosphosites, including 15 pS, 8 pT, and 2 pY, were identified in Tight junction protein ZO-1 (Tjp1), an important cell adhesion protein in sEVs.

At the level of phosphosite, 172 phosphosites (about 5% of phosphosites identified) in sEVs were novel (not described in two comprehensive PTM databases–PhosphositePlus and dbPTM). About 40% of these novel phosphosites were on threonine and tyrosine (Fig. 3C). Alix (programmed cell death 6-interacting protein), a frequently used marker of sEVs, was identified with 13 phosphosites, among which four phosphothreonine sites were not reported previously (Fig. 3D). Since Alix is an accessory ESCRT protein and plays some roles in ESCRT-mediated protein sorting [58], these novel phosphothreonine sites on Alix might provide us some clues about its role in sEVs.

Phosphorylation pattern of sEVs was considerably different from that of cells. The sEVs-derived phosphoproteome had a high level of tyrosine (Y)-phosphorylated sites (2.89% vs 1.06% in cellular phosphoproteome) (Fig. 3E). Reactome pathway analysis of these tyrosine-phosphorylated proteins revealed that they were enriched in EPH-Ephrin signaling pathway (Fig. 3F), which is consistent with previous report [59]. An integrated EPH-Ephrin signaling network in sEVs, including tyrosine-phosphoproteins, tyrosine kinases predicted from GPS5.0, and phosphorylation sites, was constructed from STRING (Figure S3B).

In summary, phosphorylation pattern of sEVs differs from that of cells. However, this phosphorylation pattern is in agreement with sEVs from other sources, suggesting that phosphorylation on tyrosine plays some roles in the formation and function of sEVs.

### *N*-glycoproteomic analysis of sEVs from C2C12 myoblasts

**Fig. 4:**
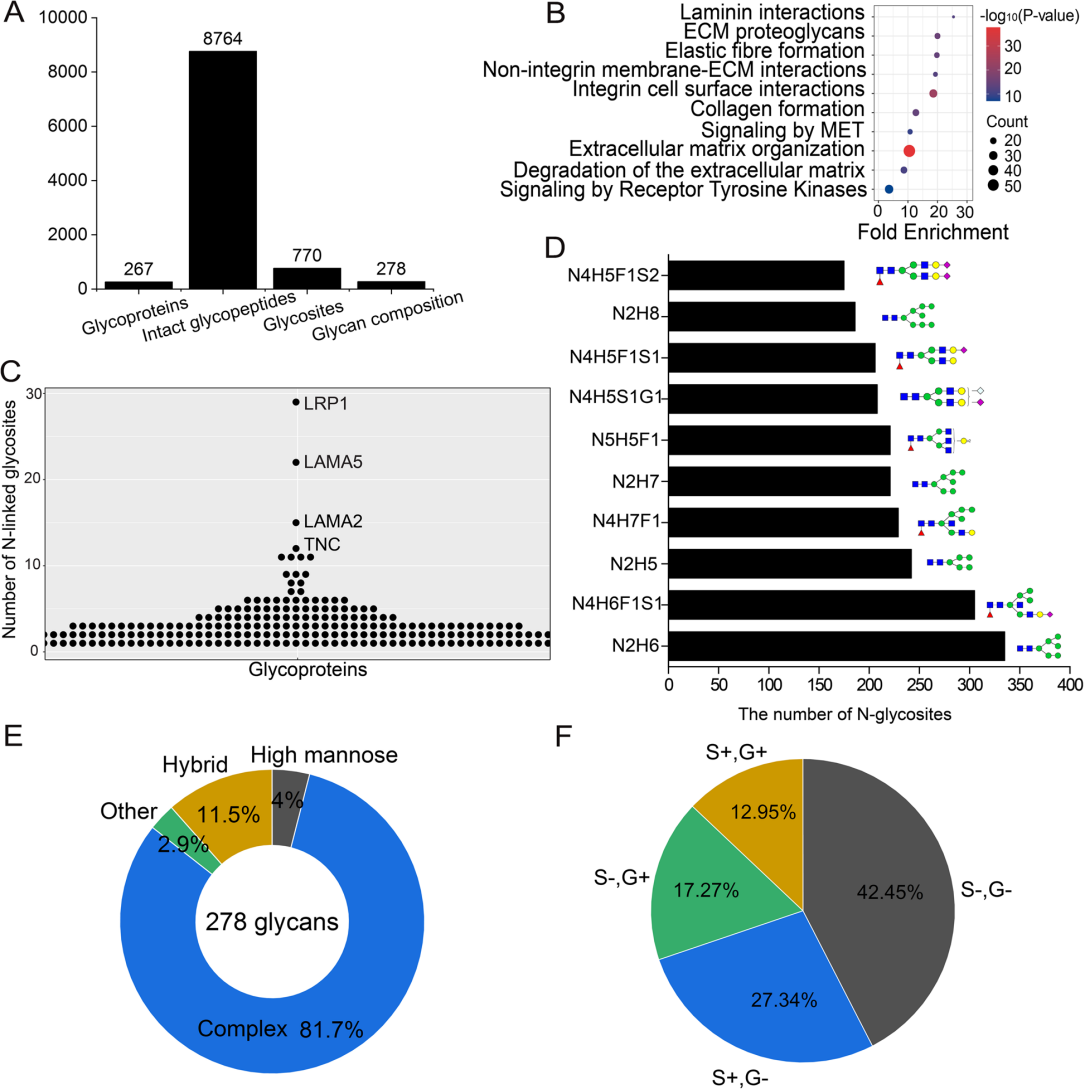
*N*-glycoproteomic analysis of C2C12 myoblasts-derived sEVs. **A** The number of *N*-glycoproteins, intact *N*-glycopeptides, *N*-glycosites, and glycan composition identified in sEVs. **B** Reactome pathway analysis of *N*-glycoproteome of sEVs. **C** The distribution of the number of *N*-glycosites on glycoproteins in sEVs. **D** Top10 *N*-glycans detected on glycoproteins of sEVs based on the numbers of their modified *N*-glycosites. **E** Distribution of *N*-glycan subtypes from intact glycopeptides identified in sEVs. **F** Distribution of *N*-glycans with or without sialic acids (S and G). S, N-Acetylneuraminic acid (Neu5Ac); **G**, N-Glycolylneuraminic acid (Neu5Gc)

*N*-Glycoproteins decorate cell surface and are released in the extracellular milieu, and play an essential role in cell-to-cell communication. It has reported that EVs are enriched in glycoconjugates (glycoproteins, glycosphingolipids, and protepglycans) and exhibit specific glycosignature [23]. In this study, we conducted *N*-glycoproteomic analysis of sEVs by enrichment of *N*-glycopeptides with ZIC-HILIC. From six LC–MS/MS analyses of *N*-glycopeptides, we documented a total number of 8764 intact *N*-glycopeptides composed of 770 *N*-glycosites and 278 glycan composition from 267 *N*-glycoproteins in sEVs of C2C12 myoblasts (Fig. 4A; Table S5).

First, we performed GO analysis of *N*-glycoproteins of sEVs (Figure S4A). GOCC analysis showed that *N*-glycoproteins were mainly from cell surface, membrane, extracellular space, extracellular region, and basement membrane. Biological processes predominantly associated with *N*-glycoproteins were cell adhesion, cell–matrix adhesion, and cell migration, indicating that *N*-glycoproteins in sEVs mainly participate in cell adhesion and cell migration. Certain binding activities, including integrin binding, collagen binding, laminin binding, calcium binding, receptor binding, were significantly overrepresented in *N*-glycoproteome of sEVs. Reactome pathway analysis revealed that several pathways, including signaling by receptor tyrosine kinases, extracellular matrix organization, signaling by MET, integrin interactions, and laminin interactions were overrepresented in *N*-glycoproteome of sEVs (Fig. 4B).

Detailed analysis of *N*-glycoproteins in sEVs revealed that more than 60% *N*-glycoproteins contained at least two *N*-glycosites (Figure S4B). On average, three *N*-glycosites were identified on the glycosylated sEV proteins. Some important proteins, which mediate the attachment, migration and organization of cells, were identified as heavily-glycosylated proteins. 29, 22, and 15 *N*-glycosites were identified on Prolow-density lipoprotein receptor-related protein 1 (LRP1), laminin subunit alpha 5 (LAMA5), and laminin subunit alpha 2 (LAMA2), respectively (Fig. 4C), which suggests glycosylation on proteins in sEVs plays important role in cell adhesion and migration.

At the level of glycoform, 6474 glycoforms were identified on 770 *N*-glycosites of glycoproteins. About 28% *N*-glycosites had only one glycoform, while 72% *N*-glycosites had at least two glycoforms. On average, 8.4 glycoforms existed on one *N*-glycosite, indicating complex micro-heterogeneity of glycosylation on glycoproteins of sEVs (Figure S4C). Some *N*-glycoproteins in sEVs displayed a highly diverse microheterogeneity. For example, 119 glycoforms were identified on glycosite N316 of lactadherin protein (MFGE8), a peripheral surface protein that binds to phosphatidylserine in the outer leaflet of exosomes [60].

At the level of *N*-glycan, top10 glycan structures appeared at different *N*-glycosites were high-mannose glycans and complex glycans (Fig. 4D). For the 278 *N*-glycans, the majority of glycans were complex glycans (81.7%) (Fig. 4E), which is in agreement with previous observation that complex N-linked glycans serve as key determination of glycoprotein sorting into EVs[22]. Detailed analysis of the composition of glycans revealed that most of N-glycans contained at least one sialic acid: Neu5Ac (S) or Neu5Gc (G) (Fig. 4F). Sialylation plays a critical role in cell recognition, cell adhesion, and cell signaling. It has reported that sialic acids are enriched in exosomes from mesenchymal stem cells compared with cell membranes and sialic acids on exosomes promoted the interaction between exosomes and cells [61]. EV sialylation also seems to play a role during EV uptake by recipient cells [62]. We speculate that the enrichment of sialic acids in the sEVs of C2C12 myoblasts would be related to sialic acid-mediated uptake of sEVs by target cells.

The surface of EVs is enriched with glycoproteins. CD63, a widely known marker of sEVs, was identified with three *N*-glycosites (N130, N150, and N172), which is in agreement with previous result [63]. The three *N*-glycosites located on the large extracellular loop of CD63 [64] and glycosylation of CD63 plays critical role in mediating its interaction with other proteins [63, 65]. The three *N*-glycosites of CD63 displayed a high degree of glycan heterogeneity. 55 glycans existed on the three *N*-glycosites. Glycosylation micro-heterogeneity on each *N*-glycosite of CD63 is shown in Figure S4D. Besides that, CD82 was identified to be glycosylated on N157. It has reported that glycosylation of CD82 at N157 is necessary for CD82-mediated inhibition of ovarian cancer cells migration and metastasis[66]. Except CD63 and CD82, another six TSPANs including TSPAN3, TSPAN6, TSPAN9, TSPAN14, TSPAN15, and CD151 were also identified as glycoproteins.

### Integrated analysis of the three proteomes of sEVs from C2C12 myoblasts

**Fig. 5:**
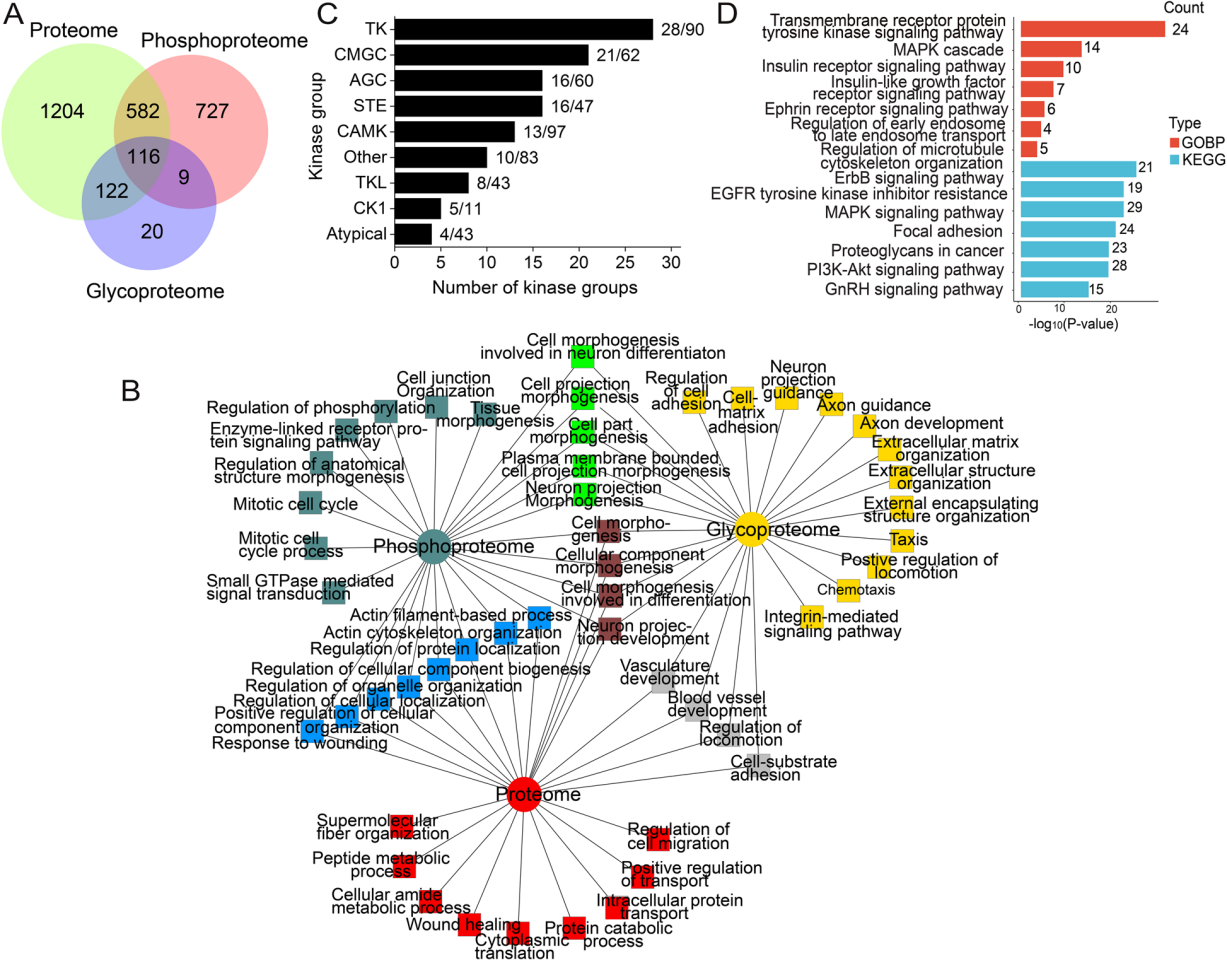
Integrated analysis of proteomic, phosphoproteomic, and *N*-glycoproteomic results of C2C12 myoblasts-derived sEVs. **A** The overlap of proteins identified in the three proteomes (proteome, phosphoproteome, and *N*-glycoproteome) of sEVs. **B** Comparative GOBP enrichment analysis of the proteome, phosphoproteome, and *N*-glycoproteome of sEVs with ToppCluster. **C** Kinase groups identified in the three-proteome dataset. **D** KEGG pathway and GOBP analysis of the kinases identified in the three-proteome dataset

We performed a comparative and integrated analysis of the three proteomes (proteome, phosphoproteome, *N*-glycoproteome) of sEVs. In total, 2780 proteins were identified in the three proteomes of C2C12 myoblast sEVs (Table S6). The overlap of proteins identified in the three proteomes is shown in Fig. 5A. 70% proteins were identified in only one proteome, suggesting that integrated proteomic and PTMomic analyses could expand the identification of cargos in sEVs. Next, we compared our dataset with Vesiclepedia database [67] (http://microvesicles.org/, accessed on 20 September 2022), which included 4937 genes of encoding exosomal proteins. 1779 proteins identified in our dataset were overlapped with Vesiclepedia database (Figure S5A) and 90 of the top100 EV proteins in Vesiclepedia database were identified in our dataset (Figure S5B), indicating a good enrichment of EV proteins.

Next, we compared our dataset with published EV proteomes of C2C12 myoblasts. In 2014, Forterre et al. [28] identified 344 proteins from EVs secreted from C2C12 myoblasts, among which 319 proteins (95.5%) were found in our results. In 2022, Watanabe et al. [29] identified 984 proteins from EVs secreted from C2C12 myoblasts, among which 795 protein (80.8%) were identified in our results. The overlap of the two published datasets with our data is shown in Figure S5C. 1985 proteins were specifically identified in our dataset, including 1244 proteins from proteome, 713 proteins from phosphoproteome, 19 proteins from *N*-glycoproteome, and 9 proteins from both phosphoproteome and *N*-glycoproteome (Figure S5C). Reactome pathway analysis of the proteins specifically identified in our data (1985 proteins) revealed that they were mainly involved in different signaling pathways, including integrin signaling, signaling by MET, signaling by EGFR, EPH-Ephrin signaling, RHOB GTPase cycle, signaling by TGFB family members, axon guidance, signaling by Rho GTPases, suggesting that integration analysis of proteomic and PTMomic data could provide more information about the role of sEVs in different signaling pathways (Figure S5D).

Comparative GOBP and GOMF enrichment analyses of the three proteomic data were conducted with ToppCluster [38], a tool for performing multi-cluster gene functional enrichment analyses. Proteins shared by all three proteomes, or two proteomes, or specifically identified in one proteome, were associated with specific biological processes (Fig. 5B). Proteins identified by all three proteomes were mainly involved in cell morphogenesis. Proteins identified in both proteome and phosphoproteome were enriched with actin filament-based process, actin cytoskeleton organization, and regulation of cellular component biogenesis. Proteins shared by proteome and *N*-glycoproteome were mainly involved in cell-substrate adhesion and regulation of locomotion, while proteins specifically identified in each proteome were enriched with unique processes, for example, proteins specifically identified in the phosphoproteome were mainly involved in signaling transduction, such as small GTPase mediated signaling transduction, enzyme-linked receptor protein signaling pathway. Proteins specifically identified in the *N*-glycoproteome were mainly involved in extracellular matrix organization, cell–matrix adhesion and integrin-mediated signaling pathway. Proteins specifically identified in the proteome were mainly involved in metabolic process and intracellular protein transport.

At GOMF level, different molecular functions were overrepresented in proteins identified in different parts. Proteins shared by the three proteomes were associated with growth factor binding, cell adhesion molecule binding, and cadherin binding, proteins identified in both proteome and phosphoproteome were mainly enriched for kinase binding, actin binding and small GTPase binding, and proteins identified in both proteome and *N*-glycoproteome were mainly involved in collagen binding, laminin binding, integrin binding (Figure S5E).

Since EVs have been reported to carry active kinases, which can be transferred to recipient cells and exert different functions through phosphorylation events [20], we focused our analysis on protein kinases detected in the three proteomic dataset. 121 kinases were identified (Table S7), among which 104 kinases were identified as phosphoproteins and 7 kinases were identified as *N*-glycoproteins. Three dominant groups of kinases identified in sEVs were: (I) tyrosine kinases, including receptor tyrosine kinase (e.g. ERBB2, ERBB3, EPHA2, EPHA7, EPHB2-4, MET, EGFR), and non-receptor tyrosine kinases (e.g. SRC, FAK, FYN, YES); (II) serine/threonine protein kinases or dual specificity kinases from CMGC group (e.g. GSK3A, GSK3B, ERK1, ERK2, CDC2, CDK2, CDK4, CDK7); (III) serine/threonine protein kinases from AGC group (e.g. AKT1, PRKACa, PRKCb) (Fig. 5C). The biological processes predominantly associated with these kinases were transmembrane receptor protein tyrosine kinases signaling pathway, MAPK cascade, and insulin receptor signaling pathway. KEGG pathways linked to these kinases were ErbB signaling pathway, EGFR tyrosine kinase inhibitor resistance, and MAPK signaling pathway (Fig. 5D), suggesting that sEVs regulate signaling pathways through these kinases.

As sEVs have reported to contain certain populations of membrane transporters to transport of substances across cells, we classified membrane transporters identified in C2C12 sEVs according to the Transporter Classification database (TCDB)[46]. 325 membrane transporters were identified in C2C12 sEV proteome and PTMomes, which mainly belonged to five classes: channels/pores, electrochemical potential-driven transporters, primary active transporters, accessory factors involved in transport, and incompletely characterized transport systems (Figure S6; Table S8). Proteins in the class of channels/pores facilitate translocation of molecules across membrane. For example, Aquaporin-1 (Aqp1) and Aquaporin-5 (Aqp5) are water channel proteins, which facilitate the transport of water across membrane. Proteins in the class of electrochemical potential-driven transporters utilize electrochemical potential to facilitate the transport of molecules across membrane. For example, some amino acid transports, including Slc1a4, Slc1a5, Slc7a5, Slc43a2, Slc38a1, mediate the uptake of amino acids across membrane. Proteins in the class of primary active transporters directly use chemical energy to transport solutes across membrane. Furthermore, about 40% of membrane transporters belong to the class of Accessory factors involved in transport, including sixteen TSPANs, such as known exosome marker proteins CD9, CD63, CD81, and CD82.

Integration of phosphorylation and glycosylation information on these membrane transports revealed that glycosylation mainly occurred on membrane transporters in the class of Accessory factors involved in transport, while phosphorylation occurred evenly on membrane transporters of the five classes (Fig. S6), which suggested that phosphorylation and glycosylation in sEVs play specific roles in transporting different substances between cells.

**Fig. 6:**
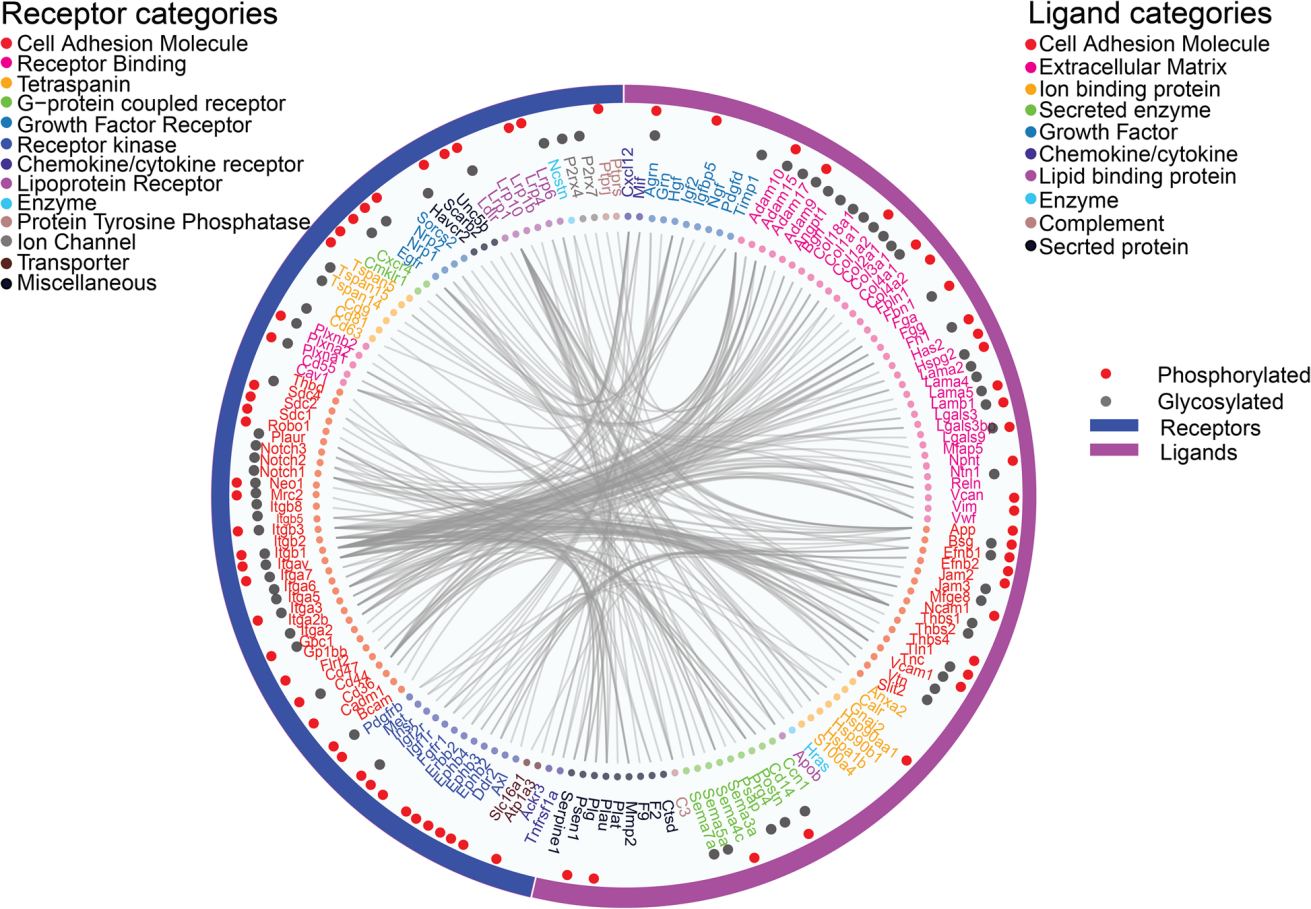
Ligand-receptor interactions identified in sEVs of C2C12 myoblasts. Ligand-receptor interactions were retrieved from CellTalk DB and constructed with R using hierarchical edge bundling. The classification of receptor or ligand categories was annotated with Uniport database. Outermost circle indicates receptors or ligands identified. Phosphorylated proteins and glycosylated proteins identified in the receptors or ligands are shown in the middle layer, with the dots color-coded. The inner layer contains gene names, color-coded for the corresponding ligand or receptor categories. Connections are marked as lines between ligands and receptors

### Ligand-receptor interaction in sEVs

As a means of intercellular communications, the “code” by which sEVs are addressed to specific recipient cells likely involves specific ligand-receptor interactions and glycoproteins [4]. To investigate intercellular communication in sEVs of C2C12 myoblasts, we sought to retrieve ligand-receptor interactions using CellTalkDB, a curated database of ligand-receptor interactions. 246 ligand-receptor pairs were retrieved in sEVs. The most enriched categories were extracellular matrix (ECM) and cell adhesion molecule (CAM) among the ligands, while CAM was enriched in the receptor categories. ECM is a complex assembly of hundreds of proteins forming the architectural scaffold of multicellular organisms, and plays an important role in cell adhesion and migration through interaction with cell-surface receptors (e.g. integrins, syndecans, adhesion GPCRs) [68]. CAM are cell-surface proteins that mediate cell-to-cell and cell-to-ECM interactions [69]. ECM and CAM represented the most abundant ligand-receptor interactions in sEVs (Fig. 6). Mapping phosphorylation and glycosylation information on these ligands and receptors revealed that glycosylation mainly occurred on ECM and CAM proteins, while phosphorylation occurred on different categories of receptors and ligands (Fig. 6 and Table S9).

**Fig. 7:**
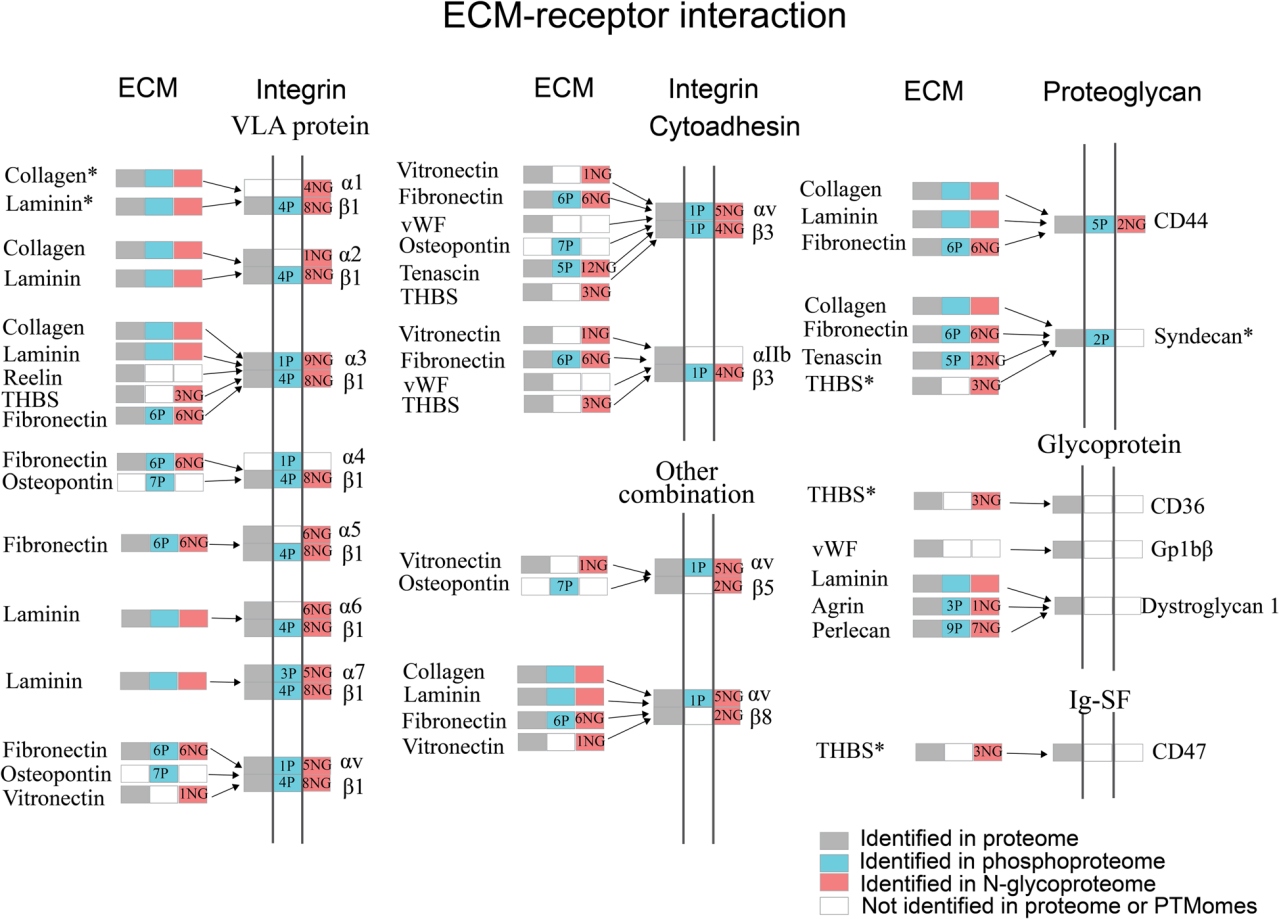
Comprehensive PTM map of ECM-receptor interaction in sEVs. ECM-receptor interaction was retrieved from Pathview. P and NG indicate phosphorylation and *N*-glycosylation, respectively. The number before P or NG indicates the number of phosphorylation sites or *N*-glycosylation sites identified on the proteins. *Collagen, laminin, syndecan, and THBS (thrombospondin) were identified with several isoforms or subunits. The detailed information of collagen isoforms and laminin subunits are shown in Figure S7 and S8, respectively

**Fig. 8:**
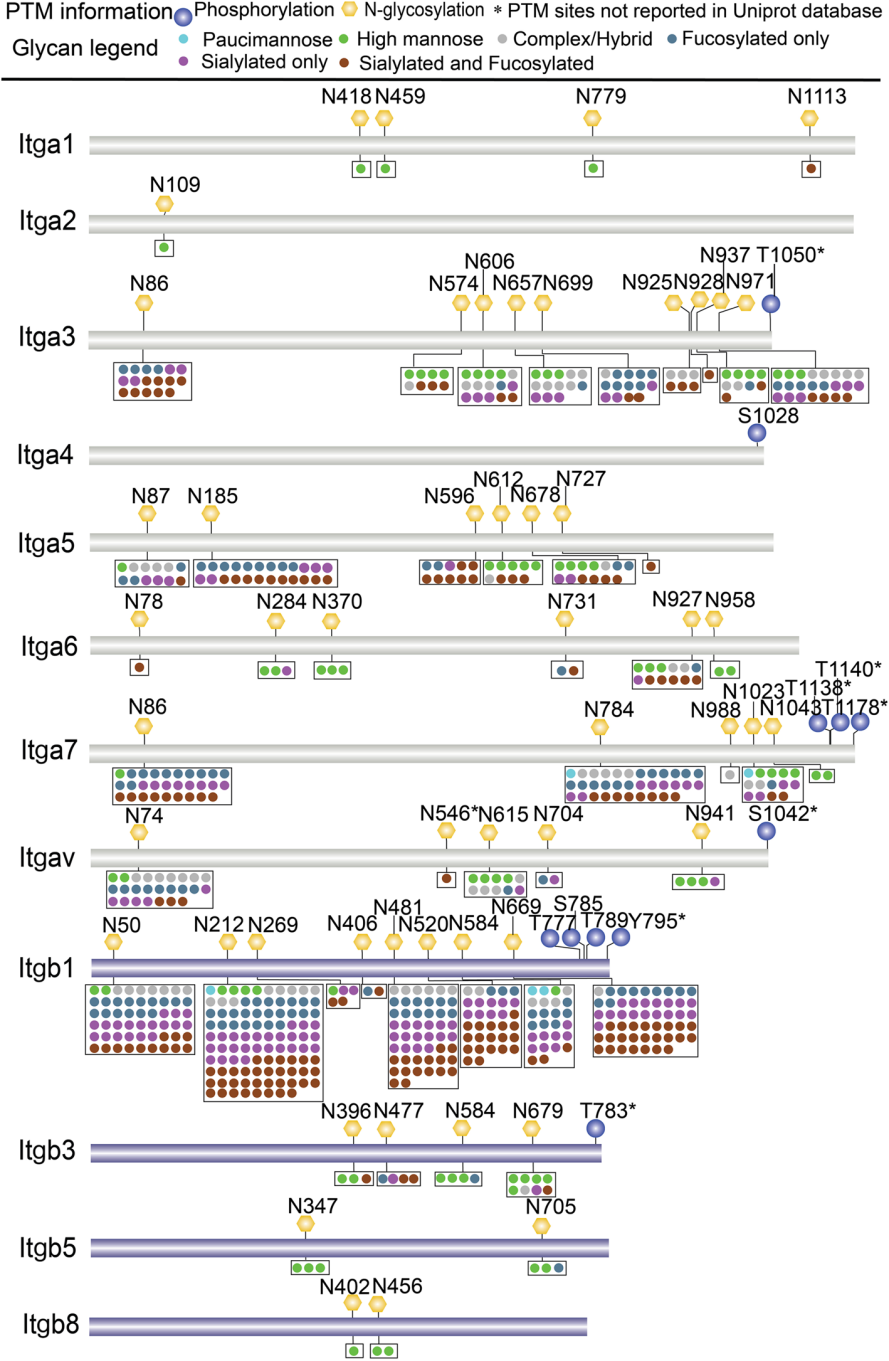
Comprehensive PTM information and glycan heterogeneity on each N-glycosite of integrin subunits identified in sEVs of C2C12 myoblasts. Itga2b (Integrin alpha-IIb) and Itgb2 (Integrin beta-2) are not displayed in the figure, as they were identified with no PTM information. Itga1, Integrin alpha-1; Itga2, Integrin alpha-2; Itga3, Integrin alpha-3; Itga4, Integrin alpha-4; Itga5, Integrin alpha-5; Itga6, Integrin alpha-6; Itga7, Integrin alpha-7; Itgav, Integrin alpha-V; Itgb1, Integrin beta-1; Itgb3, Integrin beta-3; Itgb5, Integrin beta-5; Itgb8, Integrin beta-8

Since ECM-receptor interaction is the most abundant ligand-receptor interactions in sEVs, a comprehensive map of ECM-receptor interaction was retrieved with Pathview (Fig. 7). Most ECM proteins and receptors underwent extensive phosphorylation and/or *N*-glycosylation modifications. Receptors in sEVs, mainly different integrin subunits, were identified to be phosphorylated and/or *N*-glycosylated. Integrins are a large family of heterodimeric transmembrane receptors comprising α and β subunits. The extracellular domain of integrin subunits associate with ECM proteins, while cytoplasmic domain of intergins acts as both a receptor and transmitter of signals by binding with many cellular signaling molecules. In this way, integrins constitute both a structural connection and a bi-directional signaling pathway that crosses cell membrane [70, 71]. Intergins play important role in the interaction of cells with each other and with ECM [72]. In this study, 14 integrin subunits were identified in sEVs of C2C12 myoblasts, among which 6 subunits were phosphorylated and 11 subunits were glycosylated. A comprehensive PTM map of different integrin subunits including their phosphorylation sites, *N*-glycosites and glycan heterogeneity on each glycosite is shown Fig. 8. It is intriguing that all phosphorylation sites are at the end of integrins, suggesting these phosphosites may be important for sending cellular signaling into the cells. *N*-glycosylation is essential for integrin heterodimerization, stabilization of conformation, expression at the cell membrane, and interaction with ligands [72]. Here, we observed that integrin subunits displayed different levels of glycosylation microheterogeneity. Some integrins, such as Itga1, Itga2, Itgb5, and Itgb8, had several *N*-glycosites but relatively little glycan heterogeneity overall. Some *N*-glycosites on Itga5, Itga6, Itga7, Itgav, and Itgb3 showed notably little heterogeneity while other *N*-glycosites displayed high levels of glycan microheterogeneity. All the *N*-glycosites on Itga3 and Itgb1 displayed high levels of glycan microheterogeneity. This meta-heterogeneity of glycosylation [73] on integrin subunits in sEVs might link to the specificity of the uptake of sEVs by different target cells.

Many ECM proteins, such as collagens, laminins, fibronectin, osteopontin, vitronectin, and tenascin, were identified in sEVs of C2C12 myoblasts. As the most abundant proteins in ECM, 15 collagen isoforms were identified in sEVs, among which 7 collagen isoforms were phosphorylated and 10 collagen isoforms were glycosylated. The PTM map of different collagen isoforms including their phosphorylation sites, *N*-glycosites, and glycan heterogeneity on each glycosite is shown Figure S7. Compared with integrins, most of glycosites on collagen isoforms display lower levels of glycan microheterogeneity.

Seven laminin subunits were identified in sEVs of C2C12 myoblasts, among which 4 subunits were phosphorylated and 6 subunits were glycosylated. Laminins are heavily glycosylated molecules and between 13 and 30% of their molecular weight is contributed by *N*-glycosylation [74]. Site-specific *N*-glycosylation pattern of laminin subunits is shown in Figure S8. As observed in integrins, laminin subunits displayed different levels of glycosylation microheterogeneity. Most of *N*-glycosites on Lama5 displayed high levels of glycan microheterogeneity. For Lamb1 and Lamc1, some *N*-glycosites displayed high levels of glycan microheterogeneity, while some *N*-glycosites displayed relatively little glycan heterogeneity. However, Lama4 displayed little glycan heterogeneity (Figure S8). The different glycosylation microheterogeneity on laminins might link to specific functions of laminins in cell–cell interaction and cell-ECM interactions.

Fibronectin, which is a core component of ECM and plays a key role in the assembly and remodeling of ECM [75], was identified with 6 phosphosites and 6 *N*-glycosites. Among these PTM sites, 3 phosphosites and 1 *N*-glycosite were newly identified (not included in PhosphoSitePlus and Uniprot Database).

Seven phosphosites were identified with Osteopontin, which is a major phosphoprotein of ECM and binds to a variety of cell surface integrins to stimulate cell–cell and cell-ECM adhesion [76]. Vitronectin, a glycoprotein found in ECM, was identified with 1 *N*-glycosite. Tenascin was identified with 5 phosphosites and 12 *N*-glycosites. The glycosylation of tenascin might regulate its binding capabilities [77].

Besides ECM-integrin interactions, some ECM proteins, such as collagens, fibronectin, tenascin, can interact with heparan sulfate proteoglycans including syndecans and CD44 (Fig. 7) to mediate the uptake of exosomes [78] or to promote cell motility [79]. In this study, both syndecan-1 and syndecan-2 were identified with two phosphosites, however, no *N*-glycosylation modification was identified on these two proteins, probably because heparan sulfate chains are O-glycosidically linked to a serine residue in the protein. CD44 was identified with five phosphosites and two *N*-glycosites. *N*-glycosylation of CD44 has reported to affect its interaction with hyaluronic acid [80].

In summary, some phosphorylation and *N*-glycosylation modifications were identified on the components of ligand-receptor interaction in sEVs of C2C12 myoblasts, which might account for the targeted biological effects of sEVs.

## Discussion

EVs are reported to play some roles in physiology and pathology, which are mediated through the cargos they carry, and different omics technologies have been applied to identify the cargos of sEVs. In this study, we performed a comprehensive and integrated analysis of the proteome, phosphoproteome, and *N*-glycoproteome of sEVs from C2C12 myoblasts.

In the proteome of sEVs from C2C12 myoblasts, we identified a group of cell-specific proteins that might account for cell-specific functions, such as proteins involved in MET signaling and axon guidance (Fig. 2B). MET is a receptor tyrosine kinase and triggers several downstream pathways to promote cell proliferation, growth and motility [81]. Axon guidance is a special case of cellular migration, which is regulated by many different families of ligands and receptors [82, 83]. These results suggested that proteins in sEVs from C2C12 myoblasts play specific function in cell growth and migration, which might account for the observation that EVs from C2C12 could enhance cell survival and neurite outgrowth of a motor neuron cell line [84].

In the phosphoproteome of sEVs, sEVs displayed a distinct phosphorylation pattern compared with that of cells (Fig. 3E). The high level of tyrosine-phosphorylated sites in sEVs was also observed in a few large-scale phosphoproteomic analysis of sEVs from different cell types or body fluids [59, 85, 86], suggesting that tyrosine phosphorylation of sEVs proteins may contribute to the formation and functions of sEVs [87]. Pathway analysis of the tyrosine-phosphorylated proteins indicated that they mainly participated in EPH-Ephrin signaling pathway (Fig. 3F, Figure S3B). Exosomes can mediate cell contact-independent EPH-ephrin signaling during axon guidance [88]. EPH-Ephrin signaling has been described as guidance cues that mediate migration of cells over long distances [89].In this way, phosphoproteins in sEVs of C2C12 myoblasts might mediate cell migration through EPH-Ephrin signaling pathway.

To date, most of large-scale *N*-glycoproteomic analyses of EVs have been conducted in EVs from body fluids such as urine. There is few comprehensive *N*-glycoproteomic analysis of EVs from cell culture medium, probably because of the low yield and purity of current sEV isolation approaches. Complex glycans and sialic acid-containing glycans were enriched in sEVs of C2C12 myoblasts (Fig. 4E and F). Since glyco-interaction play important role on the uptake of EVs by target cells, detailed analysis microheterogeneity of glycosylation would provide more information about the specific uptake of EVs by recipient cells.

Though different mechanisms of sEVs uptake, including ligand-receptor interaction, direct fusion with cell membrane, and endocytosis pathways, exist in the same cell [90], specific targeting of sEVs to recipient cells is determined by recognition between ligands/receptors at the surface of EVs and ligands/receptors on the plasma membrane of the recipient cells [91]. Specific enrichment of surface molecules in sEVs, mainly CAM and ECM proteins, is critical for specific uptake of sEVs. Surface glycosylation patterns is also essential for the uptake of sEVs by recipient cells.

CAM plays an important role in anchoring and internalizing exosomes [69]. CAM identified in sEVs of C2C12 myoblasts included integrins, TSPANs and glycoproteins. Based on mass spectrometric analysis, 15% of all adhesion proteins on the surface of exosomes were integrins [92, 93]. Intergins bind a diverse group of ligands, such as several ECM components (collagens, laminins, and fibronectins), and other cell receptors or soluble molecules [72]. It has reported that exosomes play crucial roles in the development of organ-specific metastases through distinct integrin expression patterns. For example, exosomal integrin α6β4 on breast cancer exosomes and integrin αvβ5 on pancreatic cancer exosomes showed an essential role in the uptake of exosomes by lung fibroblasts and liver macrophages, respectively [92]. In this study, 14 integrin isoforms were identified in C2C12 sEVs, including 9 α-and 5 β-intergin isoforms. The αβ pairings of integrin subunits dictate the specificity of integrin to a particular ligand to form intracellular adhesion complexes, and regulate downstream signaling [71]. The activity of integrins is strongly influenced by glycans through glycosylation events [72, 94]. Glycosylation of integrins affects cellular signaling and interaction with the extracellular matrix, receptor tyrosine kinases, and galectins, thereby regulating cell adhesion, motility, growth, and survival [95, 96]. Alteration of N-glycans on integrins might regulate their interactions with membrane-associated proteins, including EGFR and TSPANs [97]. In this study, site-specific *N*-glycosylation patterns were observed for different integrin subunits. Integrin subunits displayed different levels of glycosylation microheterogeneity (Fig. 8). The integrin β1 subunit displayed the highest level of mciroheterogeneity, which is consistent with the observation that β1 subunit is the most frequently seen β subunit integrin heterodimers (Fig. 7). *N*-glycosylation of different domains of integrin β1 and α5 plays crucial roles in the formation of integrin α5β1 heterodimer and its biological functions such as cell adhesion and cell migration [97–99]. Though mechanism underlines the formation of unique integrin expression pattern in sEVs still needs further investigation, different glycosylation patterns on integrins subunits might provide some insights for understanding of the roles of integins in specific targeting of sEVs to recipient cells.

The laminin-binding integrins (α3β1, α6β1, α7β1) show robust associate with TSPAN proteins. TSPAN CD151, CD81, CD9 modulate laminin binding, thus affecting integrin-dependent neurite outgrowth, cell adhesion, migration, and morphology [70].

Glycosylation can modulate the function of TSPANs [100]. It has reported that *N*-glycosylation modulated the molecular organization of CD82 and N-cadherin, which impacted in vivo trafficking of AML cells [101]. In this study, eight TSPANs were identified as glycoproteins, however, the function of glycosylation on TSPANs in sEVs of C2C12 myoblasts needs more investigation.

The specific interaction of integrins with ECM proteins, mostly fibronectin and laminin, has been shown to have important roles in ensuring that exosomes interact with the right recipients [102]. ECM proteins identified in sEVs of C2C12 myoblasts included different collagen isoforms, laminin subunits, and fibronectin. Laminins can interact with integrins and non-integrin receptors such as syndecans and α-dystroglycan to regulate cell adhesion and normal cellular functions [103]. It has reported that *N*-glycosylation of Laminin-332 is important for its association with intergins and the subsequent cellular signaling [70, 74]. Fibronectin is a critical motility-promoting cargo. The sorting of fibronectin into exosomes depends on binding to integrins [104]. *N*-glycans on fibronectin have a role in the positive regulation of cell adhesion and directed cell migration via integrin-mediated signals [105].

In summary, many proteins identified in sEVs, such as integrins, tetraspanins, laminins, and fibronectin, as well as their PTMs especially *N*-glycosylation have been implicated in the specific interaction to affect uptake of sEVs by recipient targets [106]. Integration proteomic, phosphoproteomic and *N*-glycoproteomic analysis of sEVs would provide more information about their specific uptake mechanism.

### Supplementary Information

**Supplementary Material 1.**

## Data Availability

The mass spectrometry proteomic data have been deposited to the ProteomeXchange Consortium (http://proteomecentral.proteomexchange.org) via the iProX partner repository [107] with the dataset identifier PXD047974.

## Abbreviations

EVs: Extracellular vesicles
sEVs: Small extracellular vesicles
MVs: Microvesicles
PTM: Post-translational modification
MVB: Multivesicular body
TEM: Transmission electron microscope
NTA: Nanoparticle tracking analysis
TCL: Total cell lysate
ESCRT: Endosomal sorting complexes required for transport
TMs: Transmembrane helices
TSPAN: Tetraspanin
ECM: Extracellular matrix
CAM: Cell adhesion molecule
LAMA5: Laminin subunit alpha 5
LAMA2: Laminin subunit alpha 2
