# Supplementary material for: Integrated proteomic, phosphoproteomic, and N-glycoproteomic analyses of small extracellular vesicles from C2C12 myoblasts identify specific PTM patterns in ligand-receptor interactions
Source: Cell Commun Signal. 2024 May 16;22:273. doi: 10.1186/s12964-024-01640-8 (PMC11097525; doi:10.1186/s12964-024-01640-8)
Supplement: Supplementary file 1 — Supplementary Material 1. [file 12964_2024_1640_MOESM1_ESM.zip › Integrated proteomic, phosphoproteomic, and N-glycoproteomic analyses of small extracellular vesicles_Supporting Info.docx]

**Supporting Information**

**Integrated proteomic, phosphoproteomic, and *N*-glycoproteomic analyses of small extracellular vesicles from C2C12 myoblasts identify specific PTM patterns in ligand-receptor interactions**

**Xiulan Chen^1,2,^*, Xi Song^1,2^, Jiaran Li^1^, Jifeng Wang^1^, Yumeng Yan^1^, Fuquan Yang^1,2,^***

1. Key Laboratory of Protein and Peptide Pharmaceuticals & Laboratory of Proteomics, Institute of Biophysics, Chinese Academy of Sciences, Beijing 100101, China;

2. University of Chinese Academy of Sciences, Beijing 100049, China

* Corresponding author: Xiulan Chen (chenxiulan@moon.ibp.ac.cn), Fuquan Yang (fqyang@ibp.ac.cn)

**Supplementary materials**

**Additional files:**

**Figure S1.** **Comparison of proteomic results of C2C12 cells and sEVs.** **S1A**. The overlap of proteins identified in the proteome of C2C12 cells and sEVs. **S1B**. GOCC analysis of sEVs-specific proteins. **S1C.** GOCC and GOBP analysis of proteins identified in the proteome of C2C12 cells. **S1D**. Functional annotation of the secreted proteins identified in C2C12 myoblasts sEVs with GOMF, GOBP, and Reactome pathway.

**Figure S2. Comparison of phosphoproteomic results of C2C12 cells and sEVs**. **S2A.** The overlap of phosphoproteins identified in the phosphoproteome of C2C12 cells and sEVs. **S2B.** GOCC analysis of sEVs-specific phosphoproteins. **S2C**. GOBP analysis of phosphoproteome of C2C12 cells and sEVs. **S2D**. GOCC analysis of the phosphoproteome of C2C12 cells and sEVs. **S2E.** KEGG pathway analysis of the phosphoproteome of C2C12 cells and sEVs.

**Figure S3. In-detailed analysis of phosphoproteins and tyrosine-phosphorylation signaling network in sEVs**. **(A)** Number of phosphosites observed per phosphoproteins in sEVs. (**B)** EPH-Ephrin signaling network in sEVs.

**Figure S4. Overview of *N*-glycoproteome of C2C12 myoblasts-derived sEVs**. **S4A**. GO analysis of *N*-glycoproteins of sEVs. **S4B**. *N*-glycosite distribution among glycoproteins. **S4C.** *N*-glycoform distribution among *N*-glycosites. **S4D**. Glycosylation microheterogeneity for CD63, a marker protein of sEVs.

**Figure S5. Integrated analysis of proteome, phosphoproteome and *N*-glycoproteome of C2C12 myoblasts-derived sEVs.** **S5A.** The overlap of proteins identified in the three proteomes (proteome, phosphoproteome, and *N*-glycoproteome) and Vesiclepedia database. **S5B.** The overlap of proteins identified in the three proteomes and top100 EV proteins in Vesiclepedia database. **S5C.** Comparison of three- proteome dataset with two published references of sEVs proteome of C2C12 myoblasts. **S5D**. Reactome pathway analysis of proteins specially identified in our dataset. **S5E.** Comparative GOMF enrichment analysis of the proteome, phosphoproteome, and *N*-glycoproteome of sEVs with ToppCluster.

**Fig. S6 Classification of membrane transporters identified in sEVs of C2C12 myoblasts.** Outermost circles indicate proteins identified in *N*-glycoproteome of sEVs. The middle layer indicates proteins identified in phosphoproteome of sEVs. The inner layer indicates proteins identified in proteome of sEVs.

**Figure S7. Comprehensive PTM information and glycan heterogeneity of collagen isoforms identified in sEVs**. **Col4a1** (Collagen alpha-1(IV) chain) and **Col14a1** (Collagen alpha-1(XIV) chain) were not displayed in the figure, as they were identified with no PTM information. **Col1a1**, Collagen alpha-1(I) chain; **Col1a2**, Collagen alpha-2 (I) chain; **Col2a1**, Collagen alpha-1(II) chain; **Col3a1**, Collagen alpha-1(III) chain; **Col4a2**, Collagen alpha-2(IV) chain; **Col4a3**, Collagen alpha-3(IV) chain; **Col5a1**, Collagen alpha-1(V) chain; **Col5a2**, Collagen alpha-2(V) chain; **Col6a1**, Collagen alpha-1(VI) chain; **Col6a2**, Collagen alpha-2(VI) chain; **Col12a1**, Collagen alpha-1(XII) chain; **Col16a1**, Collagen alpha-1(XVI) chain; **Col18a1**, Collagen alpha-1(XVIII) chain.

**Figure S8. Comprehensive PTM information and glycan heterogeneity of laminin subunits identified in sEVs**. **Lama1**, Laminin subunit alpha-1; **Lama2**, Laminin subunit alpha-2; **Lama4**, Laminin subunit alpha-4; **Lama5**, Laminin subunit alpha-5; **Lamb1**, Laminin subunit beta-1; **Lamb2**, Laminin subunit beta-2; **Lamc1**, Laminin subunit gamma-1.

**Table S1.** Proteomic results of sEVs derived from C2C12 myoblasts.

**Table S2.** Secreted proteins identified in the proteome of sEVs derived from C2C12 myoblasts.

**Table S3.** Membrane proteins identified in the proteome of sEVs derived from C2C12 myoblasts.

**Table S4** Phosphoproteomic results of sEVs derived from C2C12 myoblasts and python script for phosphoproteomic analysis.

**Table S5.** *N*-glycoproteomic results of sEVs derived from C2C12 myoblasts.

**Table S6.** Integrated three proteomic results of sEVs derived from C2C12 myoblasts.

**Table S7.** Kinases identified in the three proteomes of sEVs derived from C2C12 myoblasts.

**Table S8.** Membrane transporters identified in the three proteomes of sEVs derived from C2C12 myoblasts.

**Table S9**. R code and source information for ligand-receptor interaction analysis in Figure 6.


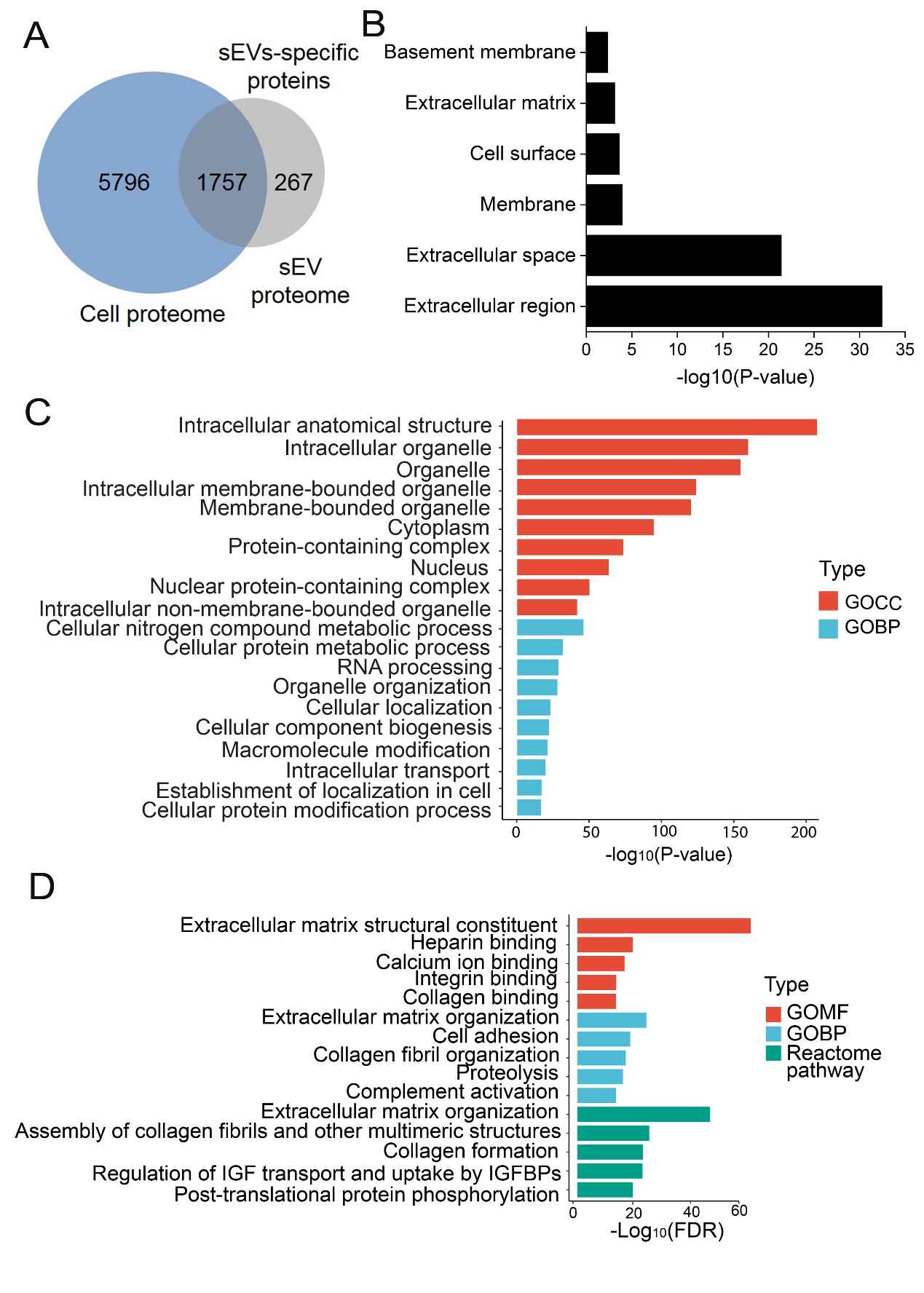


**Figure S1. Comparison of proteomic results of C2C12 cells and sEVs**. **(A)** The overlap of proteins identified in the proteome of C2C12 cells and sEVs. **(B)** GOCC analysis of sEVs-specific proteins. **(C)** GOCC and GOBP analysis of proteins identified in the proteome of C2C12 cells. **(D)** Functional annotation of the secreted proteins identified in C2C12 myoblasts sEVs with GOMF, GOBP, and Reactome pathway.


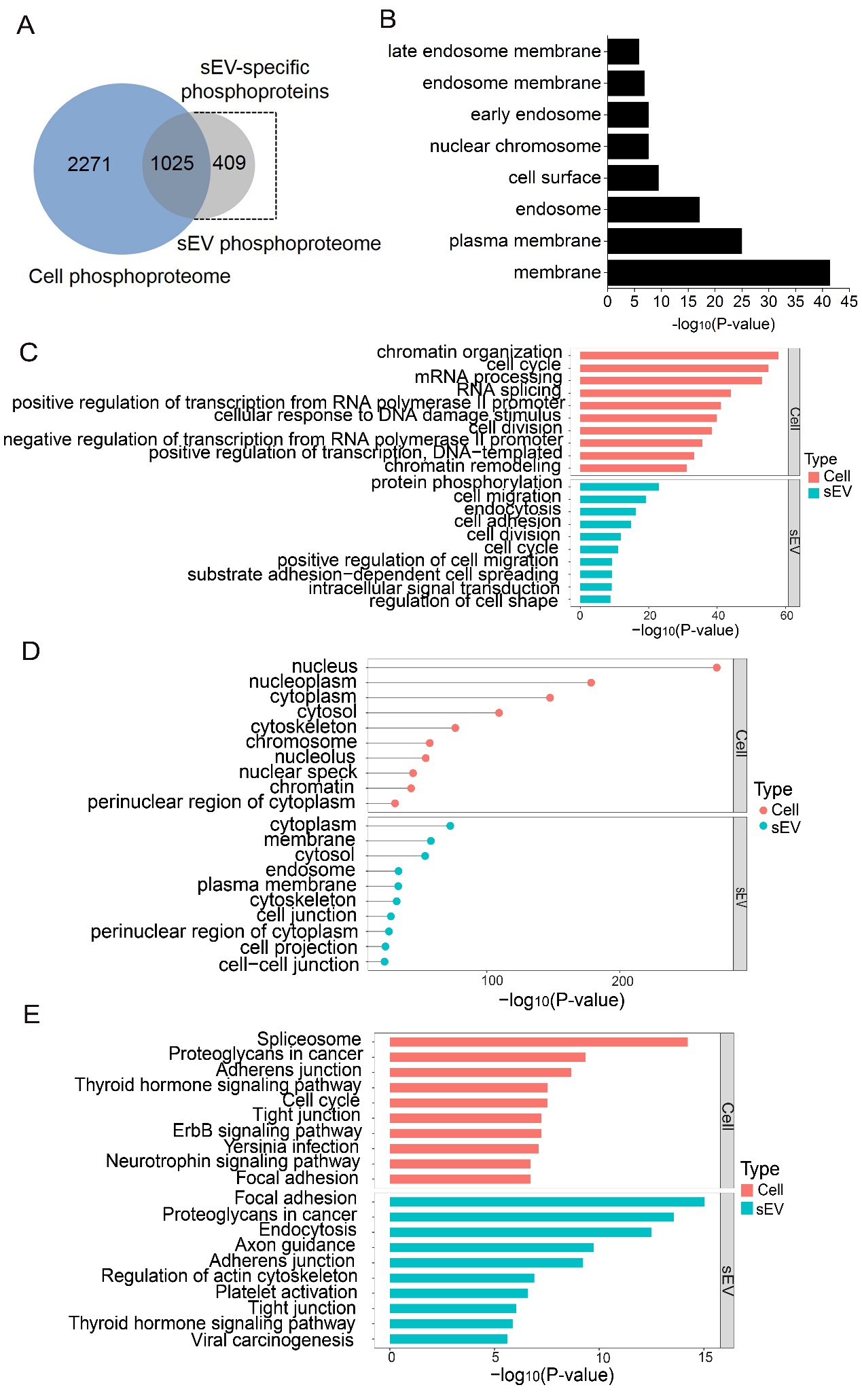


**Figure S2.** Comparison of phosphoproteomic results of C2C12 cells and sEVs. **(A)** The overlap of phosphoproteins identified in the phosphoproteome of C2C12 cells and sEVs. **(B)** GOCC analysis of sEVs-specific phosphoproteins. **(C)** GOBP analysis of phosphoproteome of C2C12 cells and sEVs. **(D)** GOCC analysis of the phosphoproteome of C2C12 cells and sEVs. **(E)** KEGG pathway analysis of the phosphoproteome of C2C12 cells and sEVs.


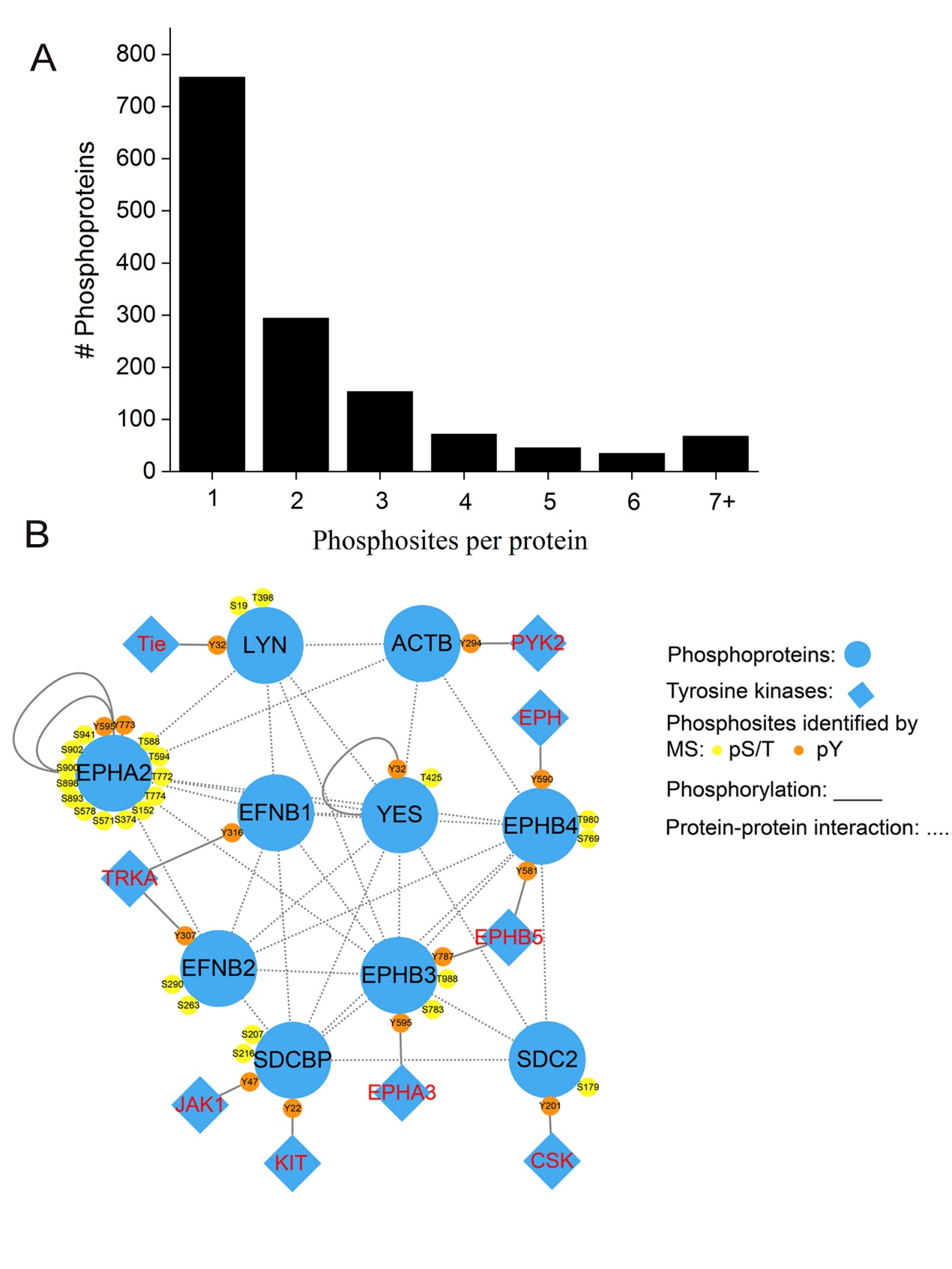


**Figure S3.** In-detailed analysis of phosphoproteins and tyrosine-phosphorylation signaling network in sEVs. **(A)** Number of phosphosites observed per phosphoproteins in sEVs. (**B)** EPH-Ephrin signaling network in sEVs.


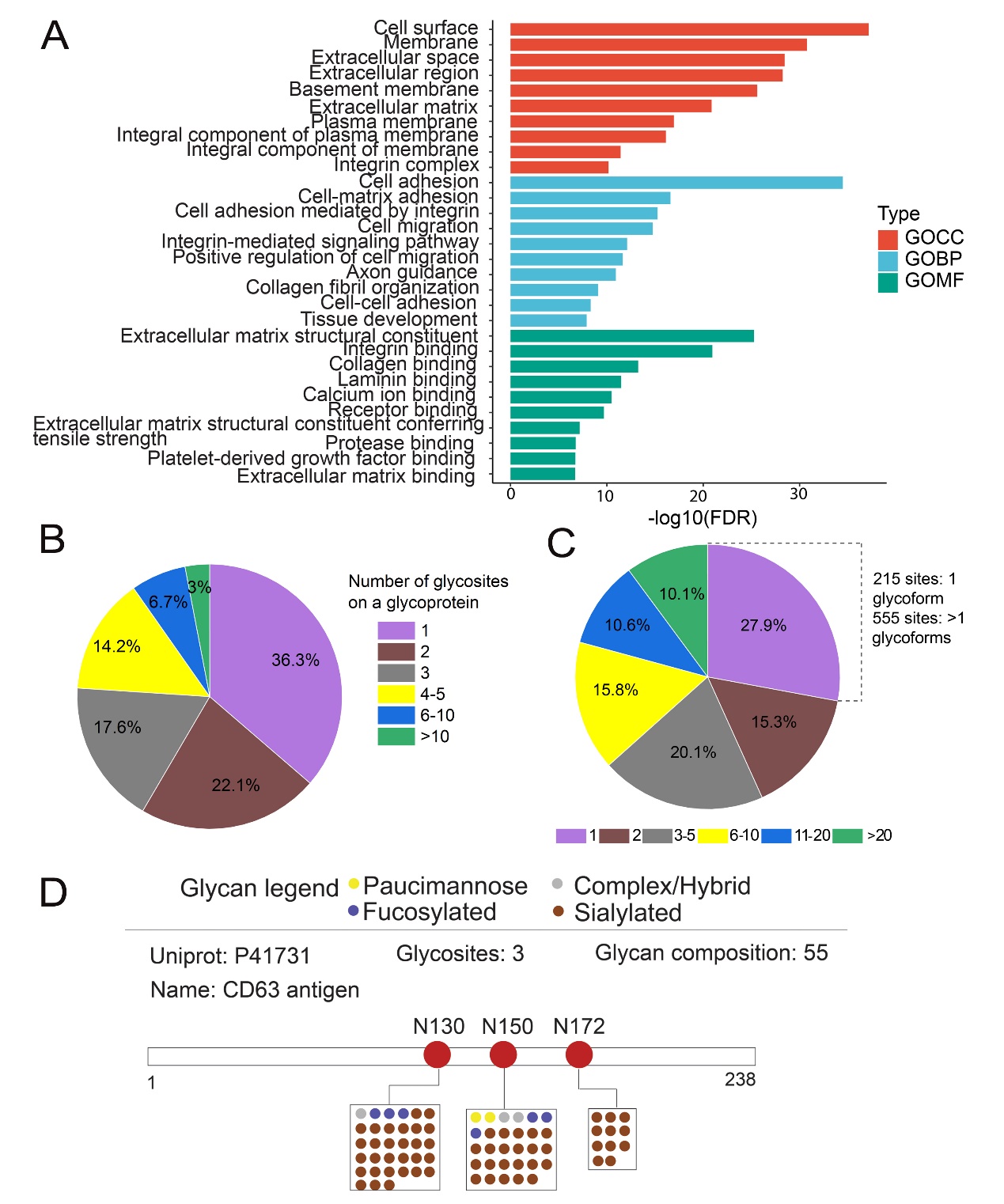


**Figure S4.** Overview of *N*-glycoproteome of C2C12 myoblasts-derived sEVs. (**A**) GO analysis of *N*-glycoproteins of sEVs. (**B**) *N*-glycosite distribution among glycoproteins. **(C)** *N*-glycoform distribution among glycosites. **(D**) Glycosylation microheterogeneity for CD63, a marker protein of sEVs.


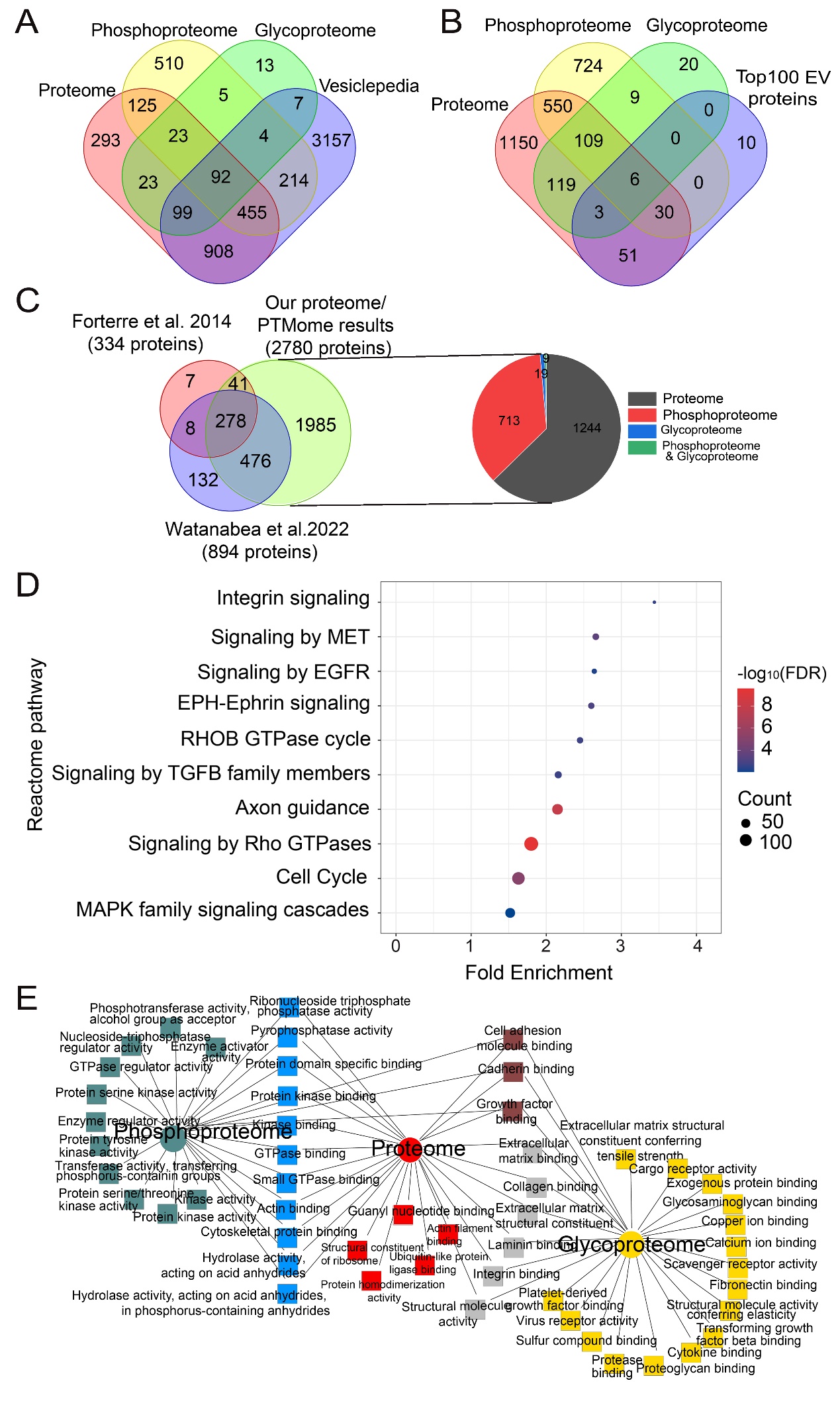


**Figure S5.** Integrated analysis of proteome, phosphoproteome and N-glycoproteome of C2C12 myoblasts-derived sEVs. (**A)** The overlap of proteins identified in the three proteomes (proteome, phosphoproteome, and N-glycoproteome) and Vesiclepedia database. (**B)** The overlap of proteins identified in the three proteomes and top100 EV proteins in Vesiclepedia database. **(C)** Comparison of three proteomic dataset with two published references about sEVs proteome of C2C12 myoblasts. **(D)** Reactome pathway analysis of the proteins specially identified in our dataset. **(E)** Comparative GOMF enrichment analysis of the proteome, phosphoproteome, and N-glycoproteome of sEVs with ToppCluster.


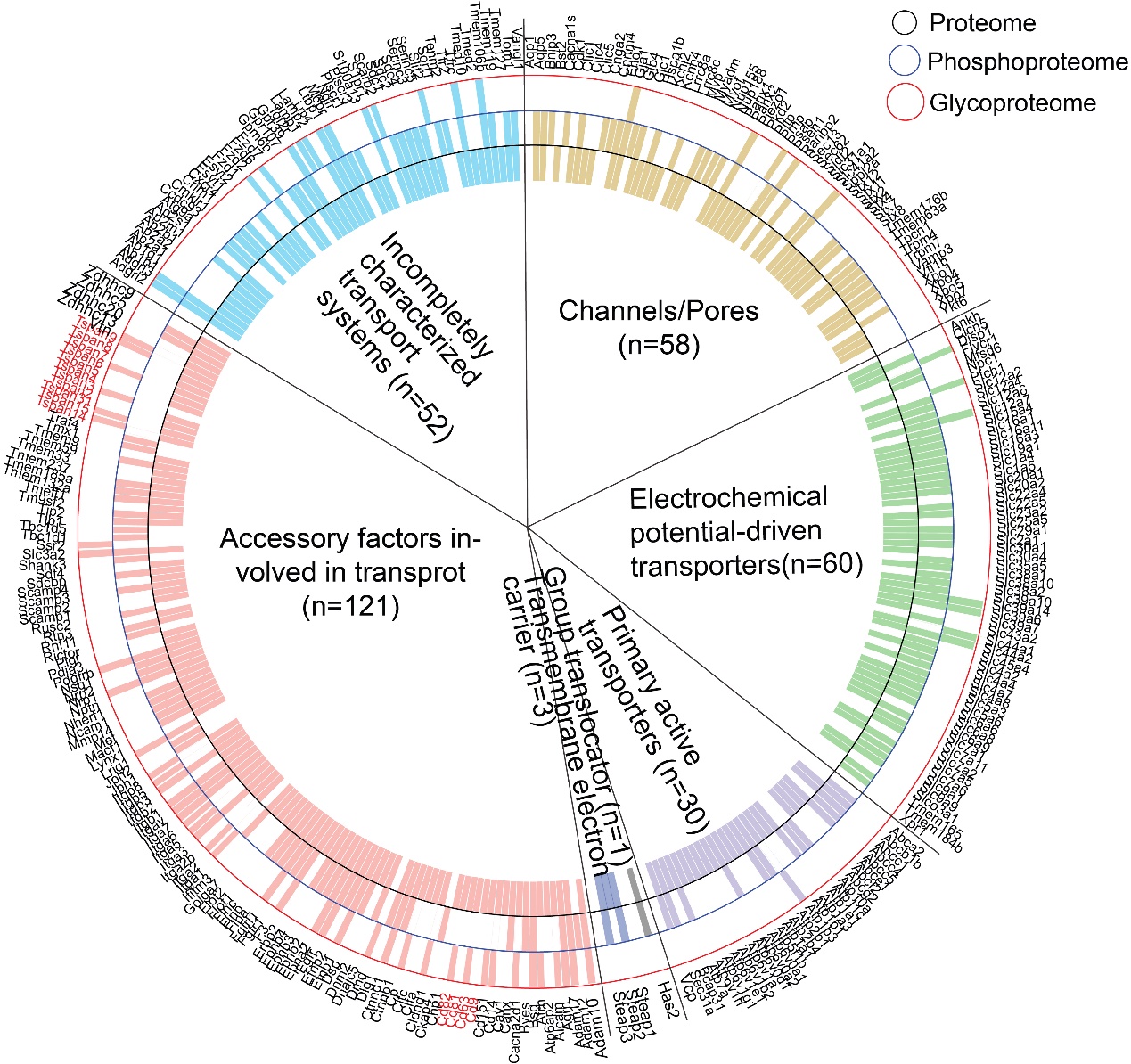


**Fig. S6 Classification of membrane transporters identified in sEVs of C2C12 myoblasts.** Outermost circles indicate proteins identified in N-glycoproteome of sEVs. The middle layer indicates proteins identified in phosphoproteome of sEVs. The inner layer indicates proteins identified in proteome of sEVs.


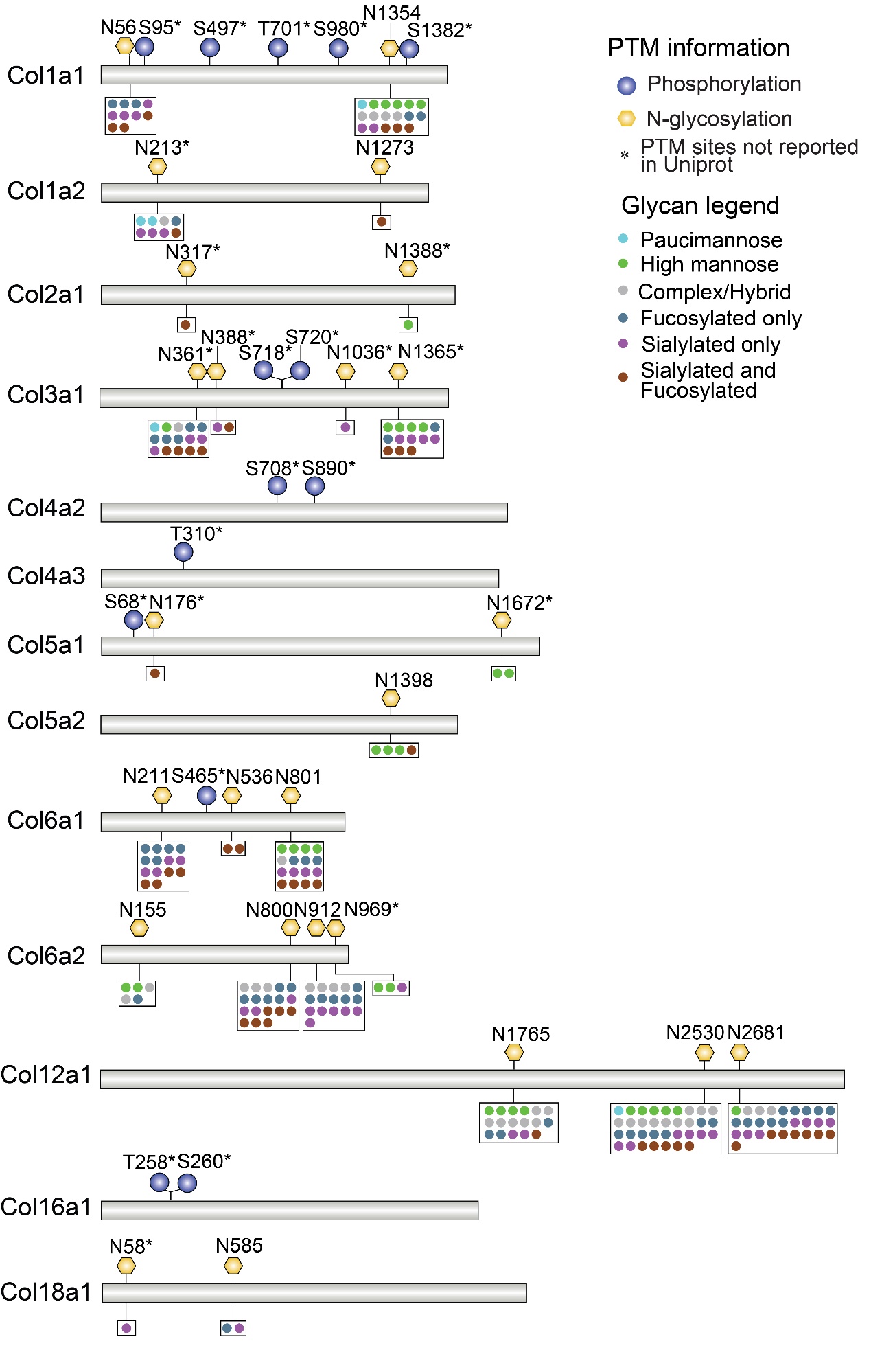


**Figure S7.** Comprehensive PTM information and glycan heterogeneity of collagen isoforms identified sEVs. Col4a1 (Collagen alpha-1(IV) chain) and Col14a1( Collagen alpha-1(XIV) chain) were not displayed in the figure, as they were identified with no PTM information. **Col1a1**, Collagen alpha-1(I) chain; **Col1a2**, Collagen alpha-2 (I) chain; **Col2a1**, Collagen alpha-1(II) chain; **Col3a1**, Collagen alpha-1(III) chain; **Col4a2**, Collagen alpha-2(IV) chain; **Col4a3**, Collagen alpha-3(IV) chain; **Col5a1**, Collagen alpha-1(V) chain; **Col5a2**, Collagen alpha-2(V) chain; **Col6a1**, Collagen alpha-1(VI) chain; **Col6a2**, Collagen alpha-2(VI) chain; **Col12a1**, Collagen alpha-1(XII) chain; **Col16a1**, Collagen alpha-1(XVI) chain; **Col18a1**, Collagen alpha-1(XVIII) chain.


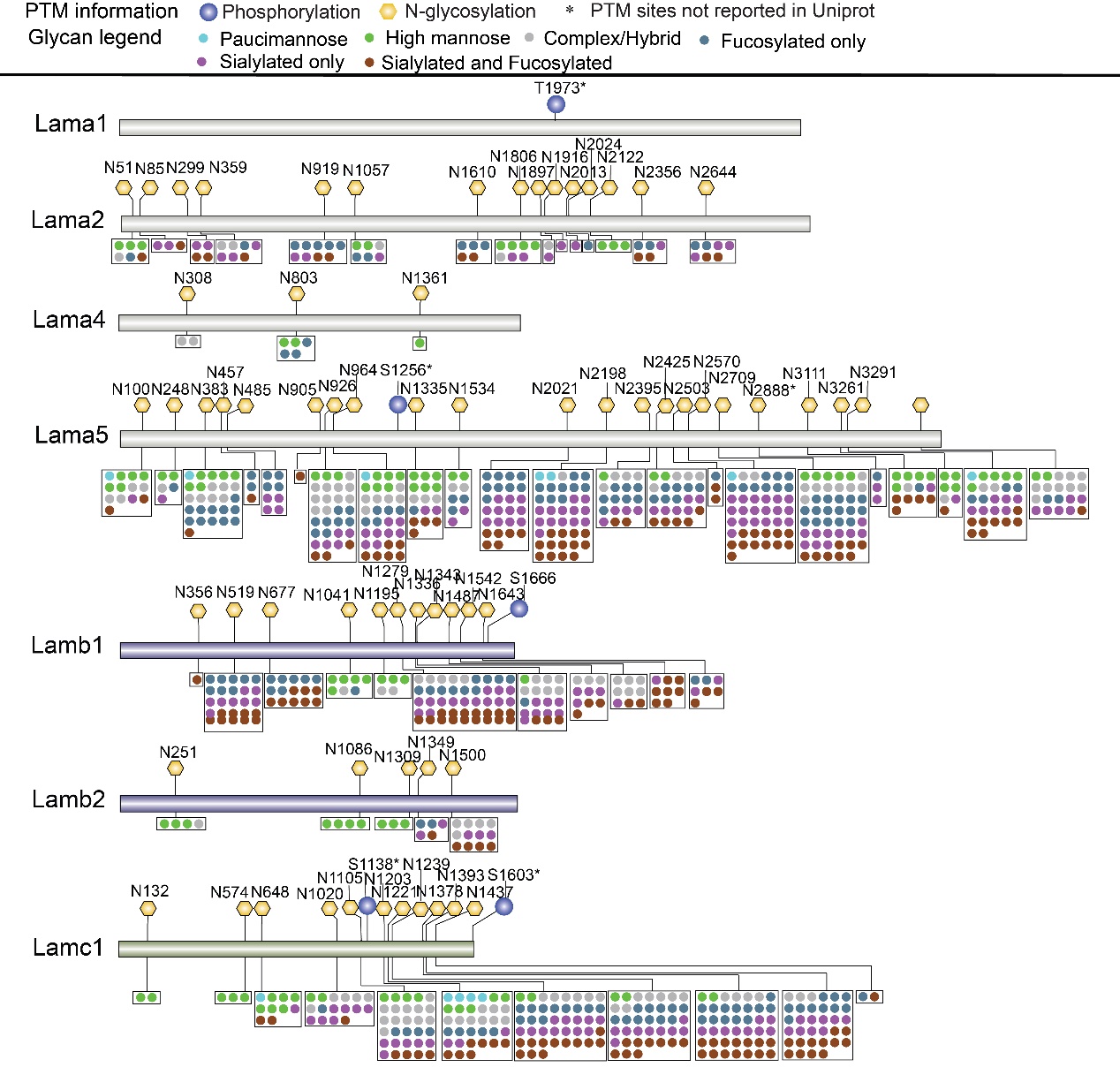


**Figure S8.** Comprehensive PTM information and glycan heterogeneity of laminin subunits identified sEVs. **Lama1**, Laminin subunit alpha-1; **Lama2**, Laminin subunit alpha-2; **Lama4**, Laminin subunit alpha-4; **Lama5**, Laminin subunit alpha-5; **Lamb1**, Laminin subunit beta-1; **Lamb2**, Laminin subunit beta-2; **Lamc1**, Laminin subunit gamma-1.
